# Exosome-derived circKIF20B suppresses gefitinib resistance and cell proliferation in non-small cell lung cancer

**DOI:** 10.1186/s12935-023-02974-y

**Published:** 2023-07-02

**Authors:** Si-Liang Wei, Jing-Jing Ye, Li Sun, Lei Hu, Yuan-Yuan Wei, Da-Wei Zhang, Meng-Meng Xu, Guang-He Fei

**Affiliations:** 1 Department of Respiratory and Critical Care Medicine, The First Affiliated Hospital of Anhui Medical University, Hefei, 230022 Anhui China; 2 Key Laboratory of Respiratory Diseases Research and Medical Transformation of Anhui Province, Hefei, 230022 Anhui China

**Keywords:** Exosome, CircKIF20B, Gefitinib resistance, Cell cycle, Apoptosis

## Abstract

**Background:**

The gefitinib resistance mechanism in non-small cell lung cancer (NSCLC) remains unclear, albeit exosomal circular RNA (circRNA) is known to possibly play a vital role in it.

**Methods:**

We employed high-throughput sequencing techniques to detect the expressions of exosomal circRNA both in gefitinib-resistant and gefitinib-sensitive cells in this study. The circKIF20B expression was determined in serum exosomes and tissues of patients by qRT-PCR. The structure, stability, and intracellular localization of circKIF20B were verified by Sanger sequencing, Ribonuclease R (RNase R)/actinomycin D (ACTD) treatments, and Fluorescence in situ hybridization (FISH). The functions of circKIF20B were investigated by 5-Ethynyl-20-deoxyuridine (EdU), flow cytometry, Cell Counting Kit-8 (CCK-8), oxygen consumption rate (OCR), and xenograft model. Co-culture experiments were performed to explore the potential ability of exosomal circKIF20B in treating gefitinib resistance. The downstream targets of circKIF20B were determined by luciferase assay, RNA pulldown, and RNA immunoprecipitation (RIP).

**Results:**

We found that circKIF20B was poorly expressed in the serum exosomes of gefitinib-resistant patients (n = 24) and the tumor tissues of patients with NSCLC (n = 85). CircKIF20B was negatively correlated with tumor size and tumor stage. Decreasing circKIF20B was found to promote gefitinib resistance by accelerating the cell cycle, inhibiting apoptosis, and enhancing mitochondrial oxidative phosphorylation (OXPHOS), whereas increasing circKIF20B was found to restore gefitinib sensitivity. Mechanistically, circKIF20B is bound to miR-615-3p for regulating the MEF2A and then altering the cell cycle, apoptosis, and mitochondrial OXPHOS. Overexpressing circKIF20B parental cells can restore sensitivity to gefitinib in the recipient cells by upregulating the exosomal circKIF20B expression.

**Conclusions:**

This study revealed a novel mechanism of circKIF20B/miR-615-3p/MEF2A signaling axis involving progression of gefitinib resistance in NSCLC. Exosomal circKIF20B is expected to be an easily accessible and alternative liquid biopsy candidate and potential therapeutic target in gefitinib-resistant NSCLC.

**Graphical Abstract:**

**Figure:**
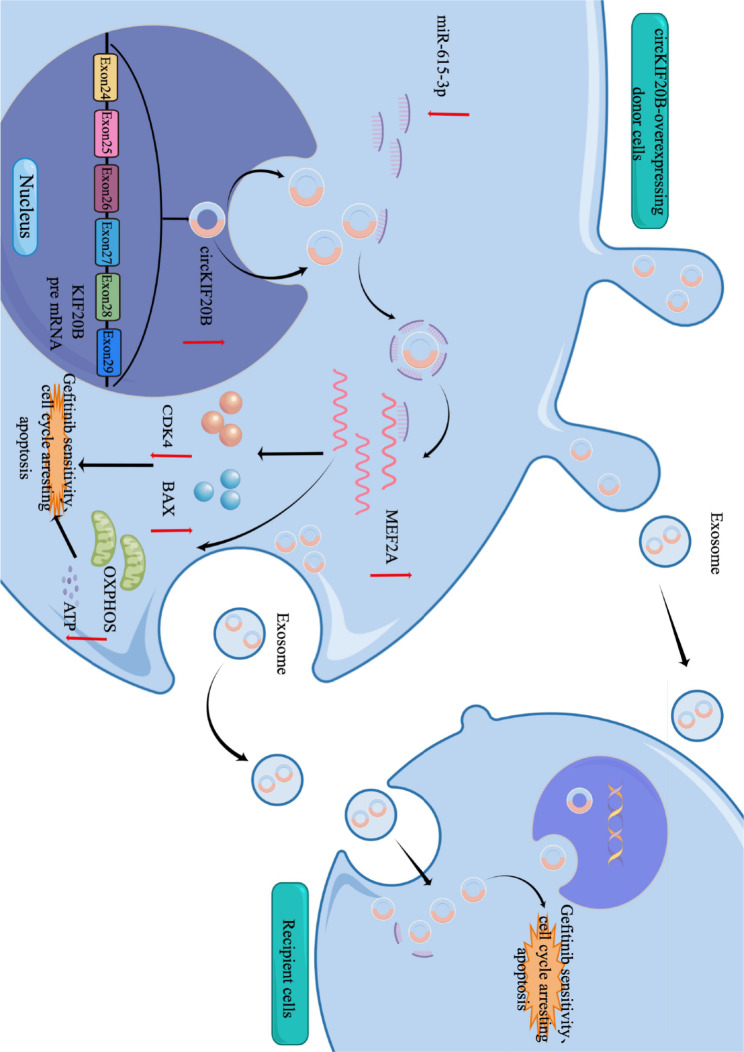
The schematic diagram of mechanism in this study. Exosomal circKIF20B inhibits gefitinib resistance and cell proliferation by arresting the cell cycle, promoting apoptosis, and reducing OXPHOS via circKIF20B/miR-615-3p/MEF2A axis in NSCLC.

**Supplementary Information:**

The online version contains supplementary material available at 10.1186/s12935-023-02974-y.

## Introduction

Globally, lung cancer ranks first as the cause of tumor-related mortality [1]. The 5-year overall survival (OS) of this disease remains ≤ 20% [2]. As one of the first-line drugs of epidermal growth factor receptor-tyrosine kinase inhibitors (EGFR-TKIs), gefitinib prolonged the survival of patients with NSCLC to a certain extent [3]. Nonetheless, several patients acquire resistance to gefitinib after taking the drug for 8–12 months [4]. Invasive diagnosis and delayed treatment of the acquired resistance often result in non-negligible harm to patients, and there is no simple mechanism to clarify the complex pathway interactions in such cases of acquired resistance. In recent years, an increasing number of studies have demonstrated that non-coding RNAs play an important role in the resistance mechanism of EGFR-TKIs, lncRNA CRNDE promoted the eIF4A3/MUC1/EGFR axis and apoptotic activity, and restored the sensitivity to EGFR-TKIs [5]. MiR-200c-3p has been reported to play a role in EGFR TKI sensitivity by regulating the EMT process [6]. Therefore, in light of the literature, new gefitinib-resistance mechanisms and effective therapeutic targets seem worth exploring.

An exosome is an extracellular vesicle of 30–150 nm [7] in diameter and is composed of a lipid bilayer. The exosome can encapsulate the genetic information of the maternal cells, including circRNA [8], long non-coding RNA (lncRNA) [9], and micro-RNA (miRNA) [10]. The exosomes deliver these RNAs to the target cells [11]. Scientists have discovered that exosomes are essential for developing drug resistance [12]. CircRNAs are non-coding RNA molecules that possess a closed circular structure [13]. In recent years, several studies have indicated that circRNA plays a significant role in the proliferation [14], metastasis [15], immune escape [16], and drug resistance [17] of malignant tumors. CircRNA is a competitive endogenous RNA (ceRNA) to regulate downstream targets by absorbing miRNA [18, 19]. MiRNAs are short non-coding RNAs of 21–22 nucleotide length [20]. CircRNA contains a specific miRNA response element (MRE) that regulates the mRNA containing the same MRE from miRNA [21]. CircRNA inhibits miRNA activity, regulates tumor-related genes, alters key pathways, and participates in the cancer process via the ceRNA network [22]. In addition, circRNAs are widely enriched in humoral exosomes [23]. We had previously reported that the upregulated circRNA PTPRA inhibits the epithelial–mesenchymal transition (EMT) and the metastatic growth of NSCLC by targeting miR-96-5p [24].

MEF2A, a DNA-binding transcription factor [25], is involved in several cellular processes, including cellular mitochondrial metabolism [26], cell growth [27], and apoptosis [28]. It has also been reported that MEF2A can directly inhibit the expression of the cell cycle genes [29] and activate the apoptosis-related pathways [30]. Cyclin-dependent kinase 4 (CDK4, a kinase that drives the G1/S progression) [31] and the BCL2-associated X (BAX, an apoptotic activator) [32] are crucial for the development and treatment of acquired resistance to gefitinib. Furthermore, although the Warburg effect suggests that cancer cells prefer to use glycolysis over the aerobic cycle [33], the mitochondrial OXPHOS function of EGFR-TKI-resistant cells is significantly increased [34]; therefore, modulation of cellular OXPHOS may be an important approach to restore gefitinib sensitivity.

In this study, we obtained the differential expression profiles of circRNAs in the exosomes from gefitinib-resistant and -sensitive cells by high-throughput sequencing. A significantly poorly expressed circRNA, circKIF20B, was screened and validated in the serum exosomes and tumor tissues of patients with NSCLC. Accordingly, we hypothesized that exosomal circKIF20B regulated gefitinib resistance by the miR-615-3p/MEF2A axis. Therefore, we explored the biological function and the specific molecular mechanism of circKIF20B in developing gefitinib-acquired resistance.

## Materials and methods

### Clinical specimens

A total of 85 pairs of NSCLC and matched normal tissues were collected from The First Affiliated Hospital of Anhui Medical University (Anhui, China). The evaluation standard of normal tissues was > 5 cm away from the tumor edge [35]. All tissues were frozen in liquid nitrogen. A total of 48 patients with NSCLC who were treated with gefitinib at The First Affiliated Hospital of Anhui Medical University were enrolled in this study. A total of 24 serum samples were sourced from patients whose disease had progressed after taking gefitinib; another half of the samples were sourced from patients who had achieved a complete response or partial response after taking the drug. We evaluated the efficacy of patients receiving gefitinib according to the solid tumor evaluation standard RECIST (Version 1.1). Patients with CR (complete remission) and PR (partial remission) are regarded as a sensitive group, and patients with PD (disease progression) are regarded as a resistant group. All serums were stored at -80℃.

All patients were diagnosed with NSCLC by professional pathologists at The First Affiliated Hospital of Anhui Medical University. The clinical pathological information has been recorded in Tables 1 and 2. This study was approved by the Medical Ethics Committee from The First Affiliated Hospital of Anhui Medical University. The experiment was conducted under the World Medical Association Declaration of Helsinki.

**Table 1:** Correlation between circKIF20B expression and clinicopathological characteristics in NSCLC patients (n = 85)

Characteristic	circKIF20B expression level(Cancer/Normal)		P-value
	Low	High	
n	43	42	
**Gender, n (%)**			0.448
Female	19 (22.4%)	23 (27.1%)	
Male	24 (28.2%)	19 (22.4%)	
**Histological type, n (%)**			0.789
Adenocarcinomas	36 (42.4%)	37 (43.5%)	
Squamous cell carcinoma	7 (8.2%)	5 (5.9%)	
**Smoking status, n (%)**			0.845
No	32 (37.6%)	33 (38.8%)	
Yes	11 (12.9%)	9 (10.6%)	
**Tumor size, n (%)**			0.012*
> 3	19 (22.4%)	7 (8.2%)	
≤ 3	24 (28.2%)	35 (41.2%)	
**Lymph node metastasis, n (%)**			0.116
No	31 (36.5%)	37 (43.5%)	
Yes	12 (14.1%)	5 (5.9%)	
**Metastasis, n (%)**			1.000
No	39 (45.9%)	38 (44.7%)	
Yes	4 (4.7%)	4 (4.7%)	
**Pathologic stage, n (%)**			0.020*
I-II	29 (34.1%)	38 (44.7%)	
III-IV	14 (16.5%)	4 (4.7%)	
**Differentiation, n (%)**			0.030*
Poor	21 (24.7%)	10 (11.8%)	
well and moderate	22 (25.9%)	32 (37.6%)	
**Age, mean ± SD**	60.37 ± 9.93	60.74 ± 11.55	0.876

**Table 2:** Correlation between serum exosomal circKIF20B expression and clinicopathological characteristics of NSCLC patients (n = 24/24)

Characteristic	Gefitinib sensitive	Gefitinib resistance	P-value
**n**	24	24	
**Gender, n (%)**			0.317
Female	4 (8.3%)	8 (16.7%)	
Male	20 (41.7%)	16 (33.3%)	
**Smoking status, n (%)**			1.000
No	19 (39.6%)	18 (37.5%)	
Yes	5 (10.4%)	6 (12.5%)	
**Tumor size, n (%)**			0.018*
> 3	10 (20.8%)	19 (39.6%)	
≤ 3	14 (29.2%)	5 (10.4%)	
**Pathologic stage, n (%)**			0.040*
IIIB	18 (37.5%)	10 (20.8%)	
IV	6 (12.5%)	14 (29.2%)	
**EGFR mutation, n (%)**			0.724
19DEL	18 (37.5%)	20 (41.7%)	
L858R	6 (12.5%)	4 (8.3%)	
**Differentiation, n (%)**			0.563
Poor	11 (22.9%)	14 (29.2%)	
Well and moderate	13 (27.1%)	10 (20.8%)	
**Age, mean ± SD**	63.62 ± 12.31	63.54 ± 9.84	0.979

### Cell culture

PC9GR and HCC827GR (gefitinib-resistant NSCLC cell lines) were generated after PC9 and HCC827 upon continuous exposure to gefitinib (M1749, Abmole, China) to step up to 20 µM concentration for at least 6 months [36]. PC9 cell line and HCC827 cell line were obtained from the Fuheng Biology in 2019 (Shanghai, China), the cell line has been authenticated by STR, and the cell line has been tested for mycoplasma contamination. DMEM or RPMI 1640 (SH30022.01, Hyclone, USA) supplemented with 10% fetal bovine serum (FBS) (10099141, Gibco, USA) and 1% penicillin and streptomycin (C0222, Beyotime, China) were used as the culture media. The plates were incubated under a 5% carbon dioxide atmosphere at 37℃. The Serum-free Media (UR51102, Umibio, China) was used before the cell extraction of the exosomes. Half maximal inhibitory concentration (IC50 value) to gefitinib of PC9, PC9GR, HCC827, and HCC827GR were calculated to determine the resistance.

### Isolation and identification of exosomes

As directed by the manufacturer’s instruction, the Quick Exosome Isolation Kit (41201ES25, Yeasen, China) was used to isolate the exosomes from the serums and cells. The exosomes for circRNA sequencing were extracted utilizing an ultracentrifuge (Beckman, USA) [37]. The morphological observation of exosomes was performed by transmission electron microscopy (TEM) (FEI, USA). Western blotting demonstrated the presence of exosomal markers, including CD63, CD9, and TSG101. Particle size measurements were performed using a nanoparticle tracking analyzer (NTA) (Malvern, UK).

### High-throughput RNA sequencing

The MiRNeasy Micro Kit (217084, Qiagen, USA) was used to extract RNA from exosomes. RNA high-throughput sequencing was performed using the Total RNA-seq (H/M/R) Library Prep Kit (NR603, Vazyme, China) and PE150 (Illumina, USA). The results of this sequencing are listed in Table S1. The selection criteria were ∣FC (Fold Change)∣>1 and P-value<0.05.

### Bioinformatics analysis

The miRNA-seq and RNA-seq were downloaded from the lung adenocarcinoma (LUAD) and lung squamous carcinoma (LUSC) projects in The Cancer Genome Atlas (TCGA). R studio (3.6.3) was used for subsequent data analyses. The websites of relevant databases in this study are listed under Table S2.

### RNA and gDNA extraction, and real-time quantitative PCR (qRT-PCR)

TRIzol (15596,018, Ambion, USA) was used to isolate total RNAs from the cells, tissues, and exosomes. Tiagen (KG203, Beijing, China) provided the DNA extraction kit for cell genomic DNA extraction. The cDNA Synthesis SuperMix (11120ES60, Yeasen) was used to reverse transcript circRNA and mRNA into cDNA. The miRNA cDNA Synthesis Kit (R601, EnzyArtisan Biotech, China) was used to reverse transcript miRNA into cDNA. The QPCR SYBR Green Mix (11201ES08, Yeasen, China) was used for conducting qPCR. Electrophoresis was performed on 2% agarose gel after PCR. For qRT-PCR, the PCR products were quantified on the MX3000P (Agilent, USA). The primers were synthesized by the EnzyArtisan Biotech (China), and the 2^-ΔΔCt^ method was used for calculation [38]. The primer sequences are listed in Table S3.

### Subcellular RNA fractionation

The cells were seeded in a 6-well plate, and the cytoplasmic and nuclear RNAs were extracted using the PARIS Kit (AM1921, Ambion) as described previously [39] when the cells reached 80% confluence. The expression of circKIF20B was detected by qRT-PCR.

### Western blotting

We collected and quantified the total proteins with the RIPA lysis buffer and the enhanced BCA protein assay kit (P0010, Beyotime, China). Briefly, 20 µg protein from each sample was loaded into the gels. Then, 12.5% SDS-PAGE (PG113, Epizyme, China) was used to separate the total protein and transfer it onto the PVDF membranes (IPVH00010, Millipore, Germany). The membranes were then blocked for 1 h with 5% non-fat milk at room temperature. The primary antibodies, including CD9, CD63, TSG101, β-actin, BAX, CDK4, and MEF2A, were incubated at 4℃ overnight and the membranes were washed by TBST. A secondary antibody linked to HRP was used for 1 h at room temperature, and the membranes were washed thrice with TBST for 10 min each. The Tanon Chemiluminescent Imaging System (China) was used to detect the Western blotting. The brand and dilution ratios of antibodies are given in Table S4.

### Fluorescence in situ hybridization (FISH)

The circKIF20B probe was fluorescently labeled with FAM, and the miR-615-3p probe was fluorescently labeled with cy3. These probes were synthesized by Genepharma (Shanghai, China), and their sequences are listed in Table S3. Then, 2 × 10^4^ cells were seeded on a coverslip overnight. Then, PBS was used to wash the cells twice, and 4% paraformaldehyde was used to fix the cells for 15 min at room temperature. Next, the cells were treated with 0.1% Triton-X 100 for 15 min at room temperature and blocked for 30 min at 37℃ incubator. Then, the cells were hybridized with the probe in the dark overnight at 37℃ incubator for 16 h. Then, 0.1% Tween 20 was used to wash the cells for 10 min at 37℃, and 2 × saline sodium citrate was used to wash the cells at 37℃ and 60℃. Then, DAPI was used as a nuclear stain and observed under a confocal microscope (Zeiss, Germany) to image the coverslip.

### Transfection of knockdown and overexpression vectors

CircKIF20B siRNAs, knockdown vector LV3-circKIF20B, and matched negative control vector LV3-NC, the mimics and inhibitors of miR-615-3p, MEF2A’s siRNAs, the MEF2A overexpression vector pEX3-MEF2A, and matched negative control vector pEX3-NC were manufactured by the Genepharma (Shanghai, China). The circKIF20B overexpression vector GV689-circKIF20B and matched negative control vector GV689-NC were synthesized by Genechem (Shanghai, China). HEK293T cells were seeded into a 6-well plate (3471, Corning, USA) and grown to 80% confluency. Then, the abovementioned siRNAs and vectors were co-transfected with Lipofectamine 3000 reagent (L3000001, Invitrogen, USA) and Opti-MEM (Gibco) for 8 h. Fresh medium was added to the cells after removing the transfection medium. After transfection for 48 h, qRT-PCR was used to detect the transfection efficacy. The sequences are listed under Table S3.

### Construction of stably transfected cell lines

HEK293T cells were seeded in a 100-mm cell culture dish (430167, Corning, USA) and transfected with the circKIF20B knockdown or overexpression vectors and the helper plasmids (Hanbio, China) via Opti-MEM and the Lipofectamine 3000 reagent when cells reached 80% confluence [40]. The transfection medium was removed after 8 h and a fresh medium was added to the cells. After transfected for 48 h, the supernatant of cell was harvested and centrifuged at 1500 x*g* for 5 min and purified via filtration through a 0.45-µm filter (UFC810096, Millipore, USA) and concentrated by centrifugation at 4000 x*g* for 15 min. Then, the lentiviral titer was detected for the subsequent experiments. The target cells were seeded into a 6-well plate and transfected with lentivirus and 5 µg/mL polybrene. After transfection for 24 h, fresh 10% FBS/DMEM was added to continue the incubation of the target cells for 48 h. Because the lentivirus also expressed GFP, flow sorting (Beckman, USA) was used to screen the GFP-positive cells [41]. Harvesting and incubating the GFP-positive cells to obtain the stably transfected cell lines. Then, the expression of target genes was detected by qRT-PCR.

### Stability verification

For RNase R treatment, we used 10 units of RNase R (R0301, Geneseed, China) to 2.5 µg of total RNA and incubated it at 37℃ for 30 min. For ACTD treatment, the cells were simultaneously treated with 2 µg/mL ACTD (SBR00013, Sigma, Germany) for 0, 4, 8, 12, and 24 h, and the total RNA was extracted with TRIzol. The expression of circKIF20B and KIF20B was detected by qRT-PCR.

### RNA pulldown

The circKIF20B biotin-labeled probe and the control probe were synthesized by Jinkairui (China), and the sequences are listed in Table S3. Following cell lysis, the cell lysates were incubated with the probe. According to the manufacturer’s instructions, the experiment was performed using the Pierce Magnetic RNA-Protein Pull-Down Kit (20164, Thermo, USA). The total RNA was extracted with TRIzol from the complex to generate biotin-labeled complementary RNA targets, followed by the preparation of RNA-conjugated beads and binding and elution of RBP. The expression of circKIF20B and miR-615-3p was detected by qRT-PCR [42].

### RNA immunoprecipitation (RIP)

To analyze the level of interaction between miR-615-3p and MEF2A, the Millipore’s Magna RIP RNA Binding Protein Immunoprecipitation Kit (17–700, USA) was used to perform RIP according to the manufacturer’s protocols. The harvested cells were lysed directly. Then, 100 µL of the cell lysate was pipetted onto anti-Ago2 magnetic beads or anti-IgG magnetic beads for immunoprecipitation [43]. After washing with RIP buffer, RNA purification and qRT-PCR detected the expression of miR-615-3p and MEF2A, respectively.

### Dual-luciferase reporter gene assay

Wild-type (WT) and mutant-type (MUT) vectors of miR-615-3p MRE were synthesized by Bioogenetech (China). Then, WT or MUT vectors were co-transfected with miR-615-3p into HEK293T. The cells were then lysed and added to the reaction solution and processed using a dual-luciferase reporter gene kit (RG042S, Beyotime). The fluorescence expression was calculated using a multifunctional microplate reader (Synergy, USA). The sequences are listed in Table S3.

### Cell viability assay and IC50 value determination

For the cell viability detection, 5 × 10^3^ cells were cultured in a 96-well plate for 0, 24, 48, and 72 h with 0.05 µM or 5 µM gefitinib, respectively. For the IC50 values analyses, different concentrations of gefitinib were added to a 96-well plate and incubated for 48 h. Each well was incubated for 1 h at 37℃ with 10 µL of the Cell Counting Kit-8 (CCK-8) (C0037, Beyotime). The microplate readers (Synergy) read the absorbance at 450 nm.

### Proliferation assay (5-ethynyl-20-deoxyuridine; EdU incorporation)

The cells (2 × 10^4^) were seeded on a coverslip, and 10 µM of EdU (C0075S, Beyotime) was added, followed by incubation for 2 h at 37℃. The cells were then fixed in 4% paraformaldehyde for 15 min at room temperature, followed by permeabilization with Trixon-X 100 for 10 min at room temperature. The cells were then treated with the click reaction mixture as per the manufacturer’s instructions. Then, DAPI was used to stain the nuclear. The coverslips were photographed under a microscope (Leica, Germany).

### Cell cycle assay and apoptosis assay

The cells (1 × 10^5^) were seeded in a 12-well plate. For cell-cycle analysis, the supernatant containing dead cells was collected to terminate tryptic digestion and centrifuged at 1000 x*g* for 5 min. Then, PBS was used to wash the cells twice, and the cells were pipetted into 1 mL of ice-cold 70% ethanol at 4 ℃ overnight. Then, PBS was used to wash the cells and stained them with propidium iodide (C1052, Beyotime) for 30 min. Then, the cell cycle was analyzed by flow cytometry (Beckman). For apoptosis, the supernatant was collected to terminate tryptic digestion and centrifuged at 1000 x*g* for 5 min, followed by washing with PBS. The proportion of apoptotic cells was detected by the Annexin V-APC/7-AAD (40310ES50, Yeasen) staining [44]. All analyses were performed by flow cytometry (Beckman) as per the manufacturer’s instructions.

### Exosome labeling and tracing

The exosomes were stained with 5 µM of 3,3-dioctadecyloxacarbocyanine perchlorate (DiO) (40725ES10, Yeasen) and incubated at 37℃ for 20 min. The exosomes were filtered through a 0.22-µM filter and then added to the cell culture medium for co-culturing with the cells for 48 h [45]. A microscope (Leica) was then used to detect the green fluorescence.

### ATP generation assay

The cells (2 × 10^5^) were seeded into a 6-well plate, and 200 µL of the cell lysates were added to each well, followed by centrifugation at 12,000 ×*g* for 5 min after cell lysis. After discarding the precipitation, add 100 µL of the ATP detection solution (S0027, Beyotime) was added to each sample [46]. A multifunctional microplate reader (PE, USA) was used to calculate the chemiluminescence absorbance.

#### Mitochondrial membrane potential assay

The cells (2 × 10^5^) were seeded in a cell culture dish (801001, NEST, China), to which 0.5 mL of the TMRE working solution (C2001S, Beyotime) was added, and the dish was incubated at 37℃ for 40 min [47]. As a positive control, the cells were treated with 10 µM of carbonyl cyanide m-chlorophenyl hydrazone (CCCP) for 20 min before adding tetramethylrhodamine ethyl ester (TMRE). At the end of the incubation period, the nuclei were stained with DAPI, and the images were captured using a confocal microscope (Zeiss).

### Oxygen consumption rate (OCR) assay

According to the manufacturer’s instructions, OCR was measured with the Seahorse XF 96 Analyzers (Seahorse Bio, USA) using the XF Mito Stress Test Kit (103015, Agilent, USA). A 96-well XF special cell culture plate was seeded with 2 × 10^4^ cells/well, and the sensor probe plate was hydrated with the seahorse XF calibration solution overnight at 37℃ in a CO_2_-free incubator. Then, oligomycin (1.5 µM), 3,3-dioctadecyloxacarbocyanine perchlorate (FCCP) (1.5 µM), and rotenone/antimycin A (0.5 µM) were added successively, and the cells were measured for OCR in the medium (pH: 7.4) containing 2 mM glutamine, 10 mM glucose, and 1 mM pyruvate [48].

#### Establishment of a xenograft model

Overexpressed circKIF20B PC9 (1 × 10^7^) was subcutaneously injected into the mice’s armpits. The GV689-NC was a control group. The tumor size and weight were measured every week. After 7 weeks, the mice were sacrificed, and the tumor was collected for subsequent experiments. The Anhui Medical University’s Animal Ethics Committee approved all the procedures.

#### Immunohistochemistry and hematoxylin-eosin (HE) staining

We next used 4% paraformaldehyde to fix the tissues, followed by paraffin embedding and sectioning. The cells were then treated with the primary antibodies MEF2A and Ki67 and incubated overnight at 4℃, followed by treatment with the secondary antibody at room temperature for 1 h. Subsequently, a DAB reagent was used to visualize the IHC, and hematoxylin was used for counterstaining. For HE staining, after fixing the sections, hematoxylin staining was performed for 15 min and eosin staining for 5 min. The cells were then observed under a microscope (Leica). The brand and dilution ratios of these antibodies used in this study are listed under Table S4.

### Statistical analysis

In this study, each experiment was repeated thrice. The GraphPad prism (9.0, USA), Image J (1.53e, USA), and Figdraw (China) were employed for graph drawing and processing. Statistical analysis was performed by SPSS (23.0, USA) and Excel 2016 (Microsoft, USA), and the data were presented as the means and standard deviation. A Wilcoxon rank-sum test was applied to analyze the unpaired differential expression in TCGA. Log-rank and COX regression techniques were employed to examine the survival probability. Pearson’s test was performed for correlational analyses. Student’s *t*-tests were performed to determine the differences between the groups. The difference was statistically significant at P < 0.05. (*P < 0.05, **P ≤ 0.01, ***P ≤ 0.001, ****P ≤ 0.0001).

## Results

### Circ_0019079 is inversely associated with gefitinib resistance and NSCLC progression

**Fig. 1:**
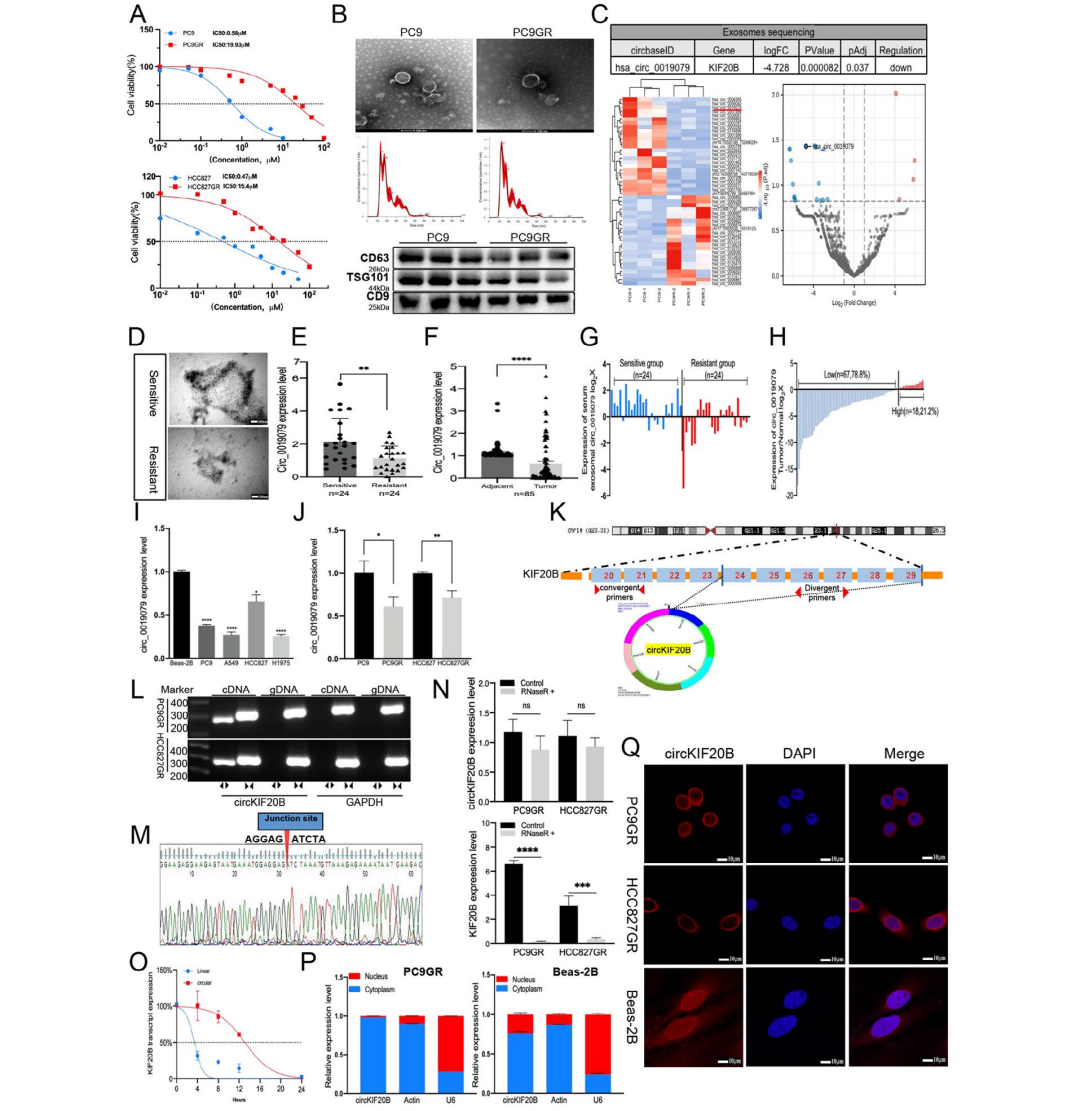
Hsa_circ_0019079 was confirmed to be a downregulated circRNA molecule in NSCLC. **A** Detection of IC50 values in PC9, PC9GR, HCC827, and HCC827GR cell lines using CCK-8 assay. **B** The exosomes of PC9 and PC9GR were extracted, and the exosomes were verified by TEM, NTA, and Western blotting. **C** The heatmap showing the significantly differentially expressed circRNAs in the exosomes from PC9 and PC9GR. The volcano plot indicates the degree of difference in the expression of circRNAs. The hsa_circ_0019079 was significantly downregulated, logFC = − 4.728, p < 0.05. **D** Electron microscopy graphs of the serum exosomes in the gefitinib-sensitive group and the gefitinib-resistant group from patients with NSCLC. **E** and **G** The relative expression of circ_0019079 in the serum exosomes of patients with gefitinib sensitivity and gefitinib resistance (n = 24), as measured by qRT-PCR with β-actin serving as the internal reference. **F** and **H** The relative expression of circ_0019079 in the NSCLC tissues and the matched normal tissues (n = 85). **I** The expression of circ_0019079 in NSCLC cell lines (PC9, HCC827, H1975, A549). A normal airway epithelial cell line (Beas-2B) as control. **J** The relative expression of circ_0019079 and mRNA KIF20B in PC9, PC9GR, HCC827, and HCC827GR. **K** Schematic representation of the formation of circKIF20B by circularization of exons 24 to 29 in the KIF20B mRNA. **L** The circular closed construction of circKIF20B was verified by divergent and convergent primers in cDNA and gDNA. **M** Sanger sequencing of circKIF20B. **N** The expression of circKIF20B and KIF20B mRNA in PC9GR and HCC827GR treated with RNase R was detected by qRT-PCR. **O** The stability of circKIF20B and KIF20B mRNA in PC9GR treated with ACTD at different times was analyzed by qRT-PCR. **P** qRT-PCR analysis of nucleoplasm fractionation products. β-actin served as an internal reference in the cytoplasm, and U6 served as an internal reference in the nucleus. **Q** Intracellular localization of circKIF20B was detected by FISH, and the nuclei were labeled with DAPI. CircKIF20B was enriched in PC9GR and the HCC827GR cytoplasm

The resistance of PC9GR and HCC827GR to gefitinib was determined by the CCK-8 assay, which revealed that the IC50 value of gefitinib in PC9GR was approximately 34-fold higher than that of PC9, and that the IC50 value of the drug in HCC827GR was approximately 32-fold higher than that in HCC827 (Fig. 1A). The exosomes were then extracted from PC9 and PC9GR and characterized by TEM, NTA, and Western blotting (Fig. 1B). After exosome identification, the high-throughput sequencing was performed to detect the differential expression of exosome-derived circRNAs. A total of 376 circRNAs were differentially expressed, of which 35 circRNAs were significantly upregulated and 341 circRNAs were significantly downregulated (screened according to the criteria of logFC > 1 or logFC < − 1, P < 0.05) (Table S1). A significantly downregulated circRNA, hsa_circ_0019079 (logFC = − 4.728, P < 0.0001) (Fig. 1C), was focused on. Serum exosomes were extracted from patients with gefitinib sensitivity and gefitinib resistance (Fig. 1D). qRT-PCR revealed that the circ_0019079 expression in the serum exosomes of the resistance group was significantly lower than that of the sensitive group (n = 24/24) (Fig. 1E and G). Circ_0019079 was also downregulated in the NSCLC tissues (n = 85) (Fig. 1F H). In 85 clinical tissue samples, the expression of circKIF20B was not significantly correlated with the patient’s age, gender, and smoking status. However, it was significantly negatively correlated with tumor size (P = 0.012), advanced tumor (P = 0.02), and Differentiation (P = 0.03). As Figure S1M shows, circKIF20B expression in stage III-IV tumors was significantly lower than in stage I-II tumors (P = 0.002). In 24 serum exosome samples, circKIF20B was negatively correlated with tumor size (p = 0.018), negatively correlated with advanced tumor (p = 0.04), and circKIF20B was not significantly correlated with age, gender, and EGFR mutation (Table 2). In the NSCLC cell lines (PC9, HCC827, A549, and H1975), the expression of circ_0019079 was significantly lower than that in the noncancer cell line (Beas-2B) (Fig. 1I). The expression of circ_0019079 in the gefitinib-resistant cell lines (PC9GR and HCC827GR) was markedly reduced than that in the sensitive cell lines (PC9 and HCC827) (Fig. 1J). These findings were consistent with the sequencing results, indicating that circ_0019079 was negatively correlated with gefitinib resistance and NSCLC progression. A search of the Circbase revealed that circ_0019079 had a spliced sequence length of 893 bp. UCSC indicated that circ_0019079 originated from the kinesin family member 20B (KIF20B) mRNA of human chromosome 10 and that it was formed by the reverse splicing of exons 24–29. This novel circRNA was named circKIF20B (Fig. 1K). The divergent primers in the.

cDNA could amplify circKIF20B, but those in the gDNA could not, thereby establishing the circular structure and reversing the spliced form of circKIF20B. Sanger sequencing confirmed the abovementioned point (Fig. 1L M). CircKIF20B exhibited more excellent resistance to RNase R than linear KIF20B (Fig. 1N). The half-life of circKIF20B was greater than that of KIF20B mRNA, as verified by ACTD treatments (Fig. 1O). Furthermore, FISH analysis (Fig. 1Q) and the nucleocytoplasmic separation qRT-PCR (Fig. 1P) revealed that circKIF20B was enriched in the cytoplasm. Collectively, the findings imply that circKIF20B is a circular closed RNA molecule that is highly stable and endogenously expressed in both the NSCLC cells and serum exosomes.

### Downregulated circKIF20B promotes NSCLC proliferation and gefitinib resistance

**Fig. 2:**
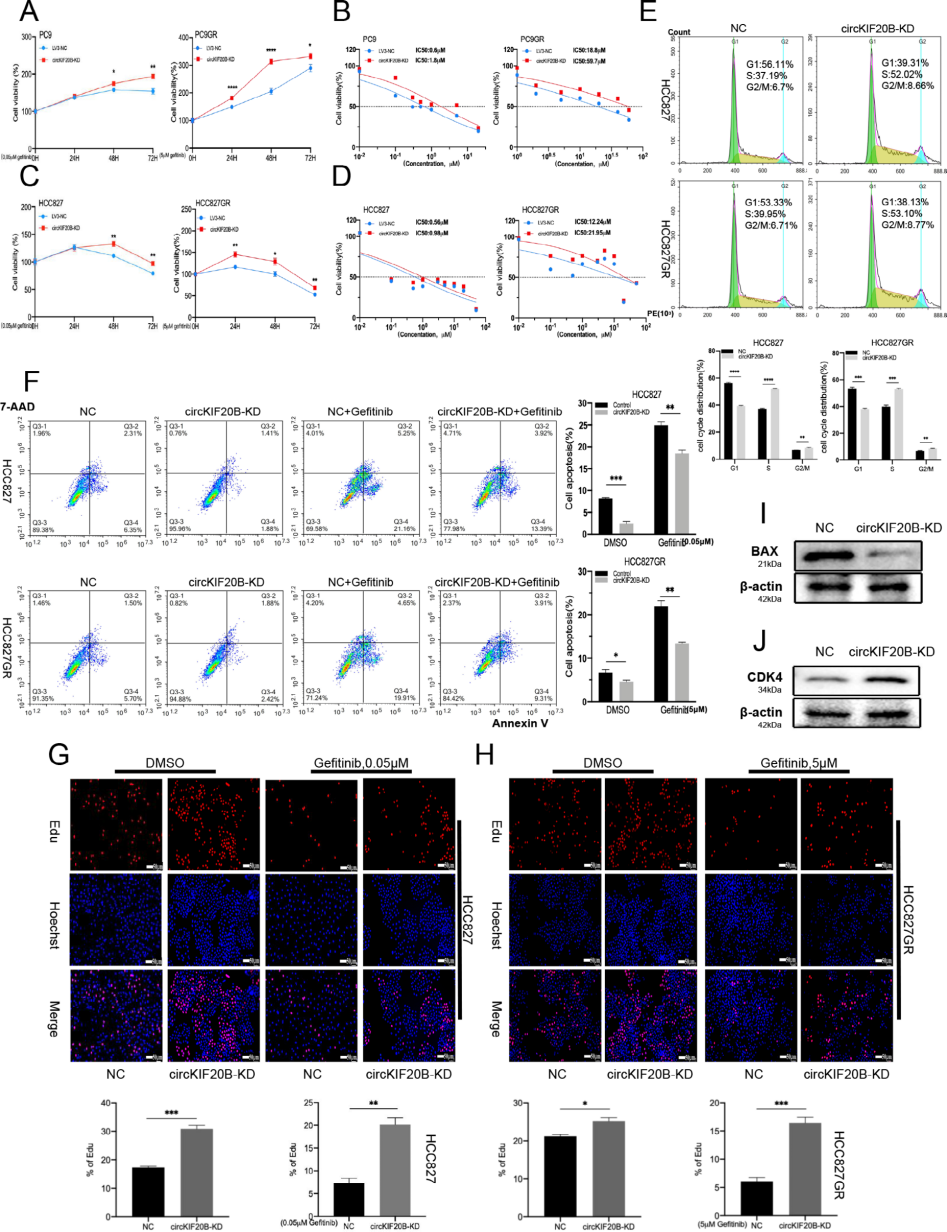
Knockdown of circKIF20B promotes gefitinib resistance and NSCLC proliferation. **A** and **C** The effect of circKIF20B knockdown on the viability of PC9, PC9GR, HCC827, and HCC827GR were analyzed by CCK-8 assay. **B** and **D** The effect of circKIF20B knockdown on the gefitinib IC50 values of PC9, PC9GR, HCC827, and HCC827GR were analyzed by CCK-8 assay. **E** The cell cycle in HCC827 and HCC827GR were detected by flow cytometry. **F** In HCC827 and HCC827GR, the apoptosis levels of the circKIF20B-KD groups were analyzed by flow cytometry in the presence or absence of gefitinib treatment. **G** and **H** In HCC827 and HCC827GR, EdU analyzed the proliferation rate of the circKIF20B-KD groups with or without gefitinib. **I** and **J** Western blotting assays verified the BAX and CDK4 protein expression in the HCC827 circKIF20B-KD group

Two circKIF20B siRNAs were designed to transfect PC9, PC9GR, HCC827, and HCC827GR. According to the qRT-PCR results, the transfection efficiency with siRNA-1 was more remarkable, and it did not affect the linear KIF20B mRNA expression (Fig. S1A and S1B). A flow sorting cytometer was employed to select the cells with a strong fluorescence expression after transfecting cells with the lentiviral vector LV3 loaded with siRNA-1 and green fluorescent protein (GFP) fragments. The selected cells were then cultured to construct stably transfected cell lines. The successful construction of the stable knockdown cell line was called circKIF20B-KD. The control group was transfected with LV3-NC. CCK-8 analysis revealed thatwhen PC9, HCC827, PC9GR, and HCC827GR have treated with gefitinib at 0.05, 0.05, 5, and 5 µM concentrations, respectively, the cell viability of the circKIF20B-KD group was significantly higher than that of the control group (Fig. 2A C). Furthermore, the knockdown of circKIF20B significantly increased the IC50 values of PC9, PC9GR, HCC827, and HCC827GR against gefitinib after simultaneous treatment of the cells with different concentrations of the drug for 48 h (Fig. 2B and D). Cell cycle assay revealed that the knockdown of cirKIF20B resulted in a decrease in the G1 phase cells and an increase in the S phase cells (Fig. 2E). Apoptotic analysis revealed a significant decrease in apoptotic cells for both HCC827 and HCC827GR in the circKIF20B-KD group compared with that in the control group. Similarly, in the case of gefitinib treatment, the knockdown of circKIF20B inhibited cell apoptosis (Fig. 2F). EdU analysis showed that, with or without gefitinib treatment, downregulated circKIF20B significantly enhanced the DNA replication rate and cell proliferation in HCC827 and HCC827GR (Fig. 2G H). Western blotting also indicated a consistent trend, that is, BAX was decreased, and CDK4 was increased in the circKIF20B-KD group of HCC827 (Fig. 2I J). Collectively, these results signify that the knockdown of circKIF20B promotes gefitinib resistance and cell proliferation in NSCLC.

### Overexpression of circKIF20B inhibits cell proliferation and gefitinib resistance in NSCLC

**Fig. 3:**
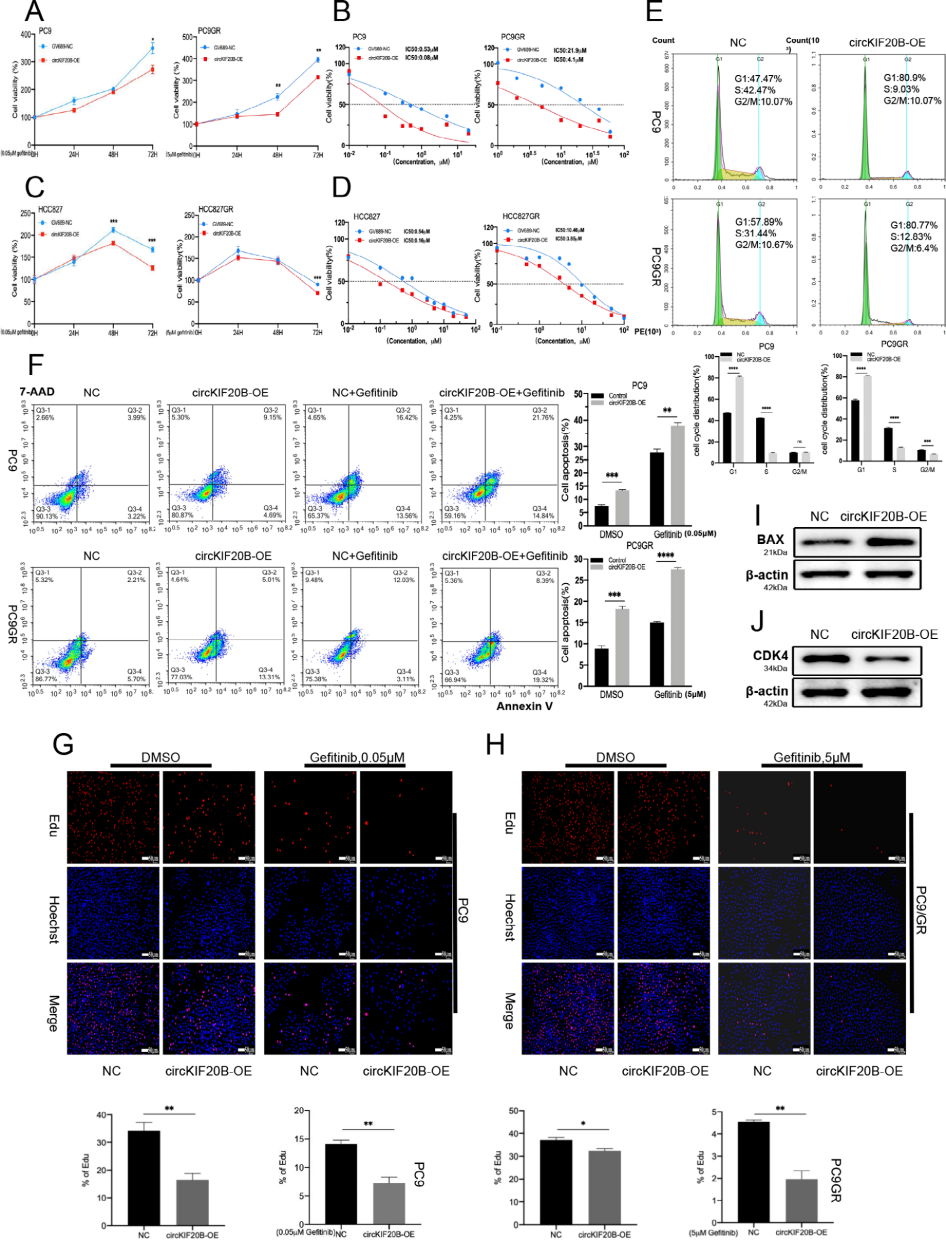
Overexpression of circKIF20B inhibits gefitinib resistance and proliferation of NSCLC. **A** and **C** The effect of circKIF20B overexpressed on the viability of PC9, PC9GR, HCC827, and HCC827GR were analyzed by CCK-8 assay. **B** and **D** The effect of circKIF20B overexpressed on the gefitinib IC50 values of PC9, PC9GR, HCC827, and HCC827GR were analyzed by CCK-8 assay. **E** With or without gefitinib, cell cycle assay of circKIF20B-OE groups was detected using flow cytometry in PC9 and PC9GR. **F** With or without gefitinib, the apoptosis level of the circKIF20B-OE groups was analyzed by flow cytometry in PC9 and PC9GR. **G** and **H** In PC9 and PC9GR, EdU analyzed the proliferation rate of circKIF20B-OE groups with or without gefitinib. **I** and **J** Western blot assays verified the BAX and CDK4 protein expression in the PC9 circKIF20B-OE group

The CCK-8 assay was performed to investigate whether circKIF20B can be an effective diagnostic and treatment target for the acquired resistance to gefitinib. The construction method of the circKIF20B overexpression stable transgenic cell line was the same as circKIF20B-KD group, it was named circKIF20B-OE (Fig. S1C and S1D), and the control group was transfected with GV689-NC. The results indicated that the cell viability of the circKIF20B-OE group was notably reduced when compared with that of the control group in PC9, PC9GR, HCC827, and HCC827GR (Fig. 3A C). After 48 h of treatment with different doses of gefitinib, the circKIF20B-OE group significantly reduced the IC50 values of gefitinib in the PC9, PC9GR, HCC827, and HCC827GR (Fig. 3B and D). The overexpression of circKIF20B increased the cell proportion in the G1 phase and decreased the cell proportion in the S phase, according to the results of the cell cycle assays (Fig. 3E). Moreover, the apoptotic analysis revealed that the apoptotic cells were dramatically increased in the circKIF20B-OE group than in the control group in PC9 and PC9GR (Fig. 3F). EdU assay revealed that the DNA replication rate and cell proliferation were significantly reduced in the circKIF20B-OE group in PC9 and PC9GR with or without gefitinib (Fig. 3G H). Western blotting results showed that BAX was upregulated, while CDK4 was downregulated in the circKIF20B-OE group of PC9 (Fig. 3I J). In conclusion, the overexpression of circKIF20B decreased the growth ability and gefitinib resistance of NSCLC in vitro.

### CircKIF20B acts as a molecular sponge for miR-615-3p

**Fig. 4:**
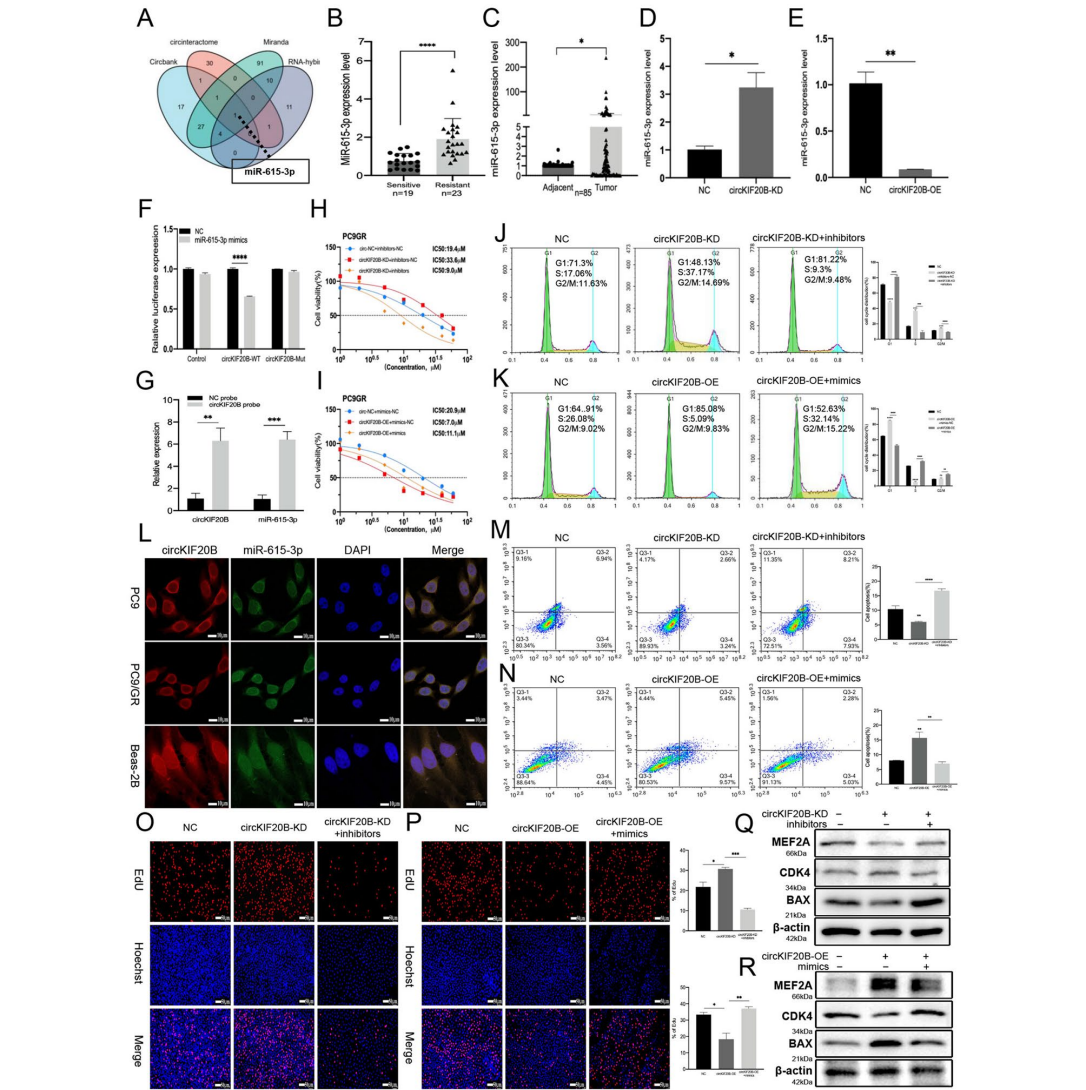
The miR-615-3p was regulated by circKIF20B to affect gefitinib IC50 value and cell proliferation. **A** MiR-615-3p was predicted as a target of circKIF20B in four databases. **B** The expression of miR-615-3p in serum exosomes of gefitinib-resistant and sensitive patients (n = 23/19), measured by qRT-PCR with U6 serving as an internal reference. **C** The expression of miR-615-3p in NSCLC tissues and matched normal tissues (n = 85). **D** The expression of miR-615-3p in the circKIF20B-KD group of PC9GR, the PC9GR transfected with LV3-NC served as control. **E** The expression of miR-615-3p in PC9GR circKIF20B-OE group, control was the cell transfected with GV689-NC. **F** The luciferase activity of HEK293T cell co-transfected with WT or MUT vectors and miR-615-3p mimics. **G** The expression of miR-615-3p was detected by qRT-PCR in PC9GR circKIF20B-OE cell lysates after pulldown with circKIF20B probe or oligo probe. The oligo probe group served as the control. **H** The gefitinib IC50 values of PC9GR co-transfected LV3-NC and inhibitors-NC, PC9GR circKIF20B-KD transfected inhibitors-NC, and PC9GR circKIF20B-KD transfected the inhibitors of miR-615-3p were detected using CCK-8 assay, respectively. **I** The gefitinib IC50 values of PC9GR co-transfected GV689-NC and mimics-NC, PC9GR circKIF20B-OE transfected mimics-NC, PC9GR circKIF20B-OE transfected the mimics of miR-615-3p were detected using CCK-8 assay, respectively. **L** The intracellular co-localization of circKIF20B and miR-615-3p in PC9, PC9GR, and Beas-2B cell lines by FISH. Nuclei were labeled with DAPI. **J** and **M** The cell cycle and apoptosis assays of PC9GR co-transfected with LV3-NC and inhibitor-NC, the circKIF20B-KD groups transfected with inhibitor-NC, and the circKIF20B-KD groups transfected with miR-615-3p inhibitors. **K** and **N** The cell cycle and apoptosis assays of PC9GR co-transfected with GV689-NC and mimic-NC, circKIF20B-OE groups transfected with mimics-NC, and the circKIF20B-OE groups transfected with miR-615-3p mimics. **O** and **P** The cell proliferation of the abovementioned groups was analyzed by EdU. **Q** and **R** The MEF2A, CDK4, and BAX protein expression of the abovementioned groups were analyzed using Western blotting

FISH analysis (Fig. 1Q) confirmed that circKIF20B was primarily distributed in the cytoplasm. Hence, it may perform biological functions through the recognized ceRNA mechanism. Circbank, Circinteratome, Miranda, and RNA-hybrid were employed to predict the downstream miRNAs of circKIF20B. MiR-615-3p was screened after taking the intersection (Fig. 4A). TCGA showed that miR-615-3p was significantly more highly expressed in the NSCLC group than in the control group (Fig. S2A and S2B). In the LUAD group, high expression of miR-615-3p was significantly associated with decreased OS of patients. In the LUSC group, there was no significant correlation (Fig. S2E and S2F), and the receiver operating characteristic (ROC) curve showed that it exhibited a significant diagnostic performance in NSCLC (Fig. S2C and S2D). In addition, the expression of miR-615-3p in the serum exosomes of patients with gefitinib resistance was significantly higher than that of patients who were sensitive to the drug (n = 19/23) (Fig. 4B). Interestingly, the qRT-PCR results of the clinical tissues were consistent with the database results (n = 85) (Fig. 4C). qRT-PCR showed that the downregulation of circKIF20B distinctly increased the expression of miR-615-3p (Fig. 4D). Conversely, the overexpression of circKIF20B significantly reduced the expression of miR-615-3p (Fig. 4E). CircRNA regulates miRNA by specific binding, which is the core mechanism of ceRNA. To further evaluate the interaction between circKIF20B and miR-615-3p, the MRE of miR-615-3p was predicted using the Circinteratome database (Fig. S2G). The MRE-binding sites of circKIF20B and miR-615-3p were confirmed with the dual-luciferase reporter assay after transfection of the HEK293T cell line with the MUT and WT vectors (Fig. 4F). Furthermore, the RNA pulldown assay demonstrated that the expression of miR-615-3p was significantly upregulated in the circKIF20B probe group than in the control group in PC9 (Fig. 4G). FISH analysis signified that miR-615-3p was colocalized with circKIF20B in the cytoplasm (Fig. 4L). After clarifying the interaction between circKIF20B and miR-615-3p, the possibility of miR-615-3p regulating gefitinib resistance was investigated. To regulate the expression of miR-615-3p, PC9GR was transfected with mimics or inhibitors of miR-615-3p, and qRT-PCR was performed to verify the effectiveness of the transfection (Fig. S1E and S1F). Subsequently, CCK-8 analysis showed that the upregulation of miR-615-3p increased the IC50 value of gefitinib, whereas the downregulation of miR-615-3p decreased it (Fig. S1K). In summary, miR-615-3p, a target of circKIF20B, plays a vital role in gefitinib resistance.

### MEF2A acts as the target gene for miR-615-3p

**Fig. 5:**
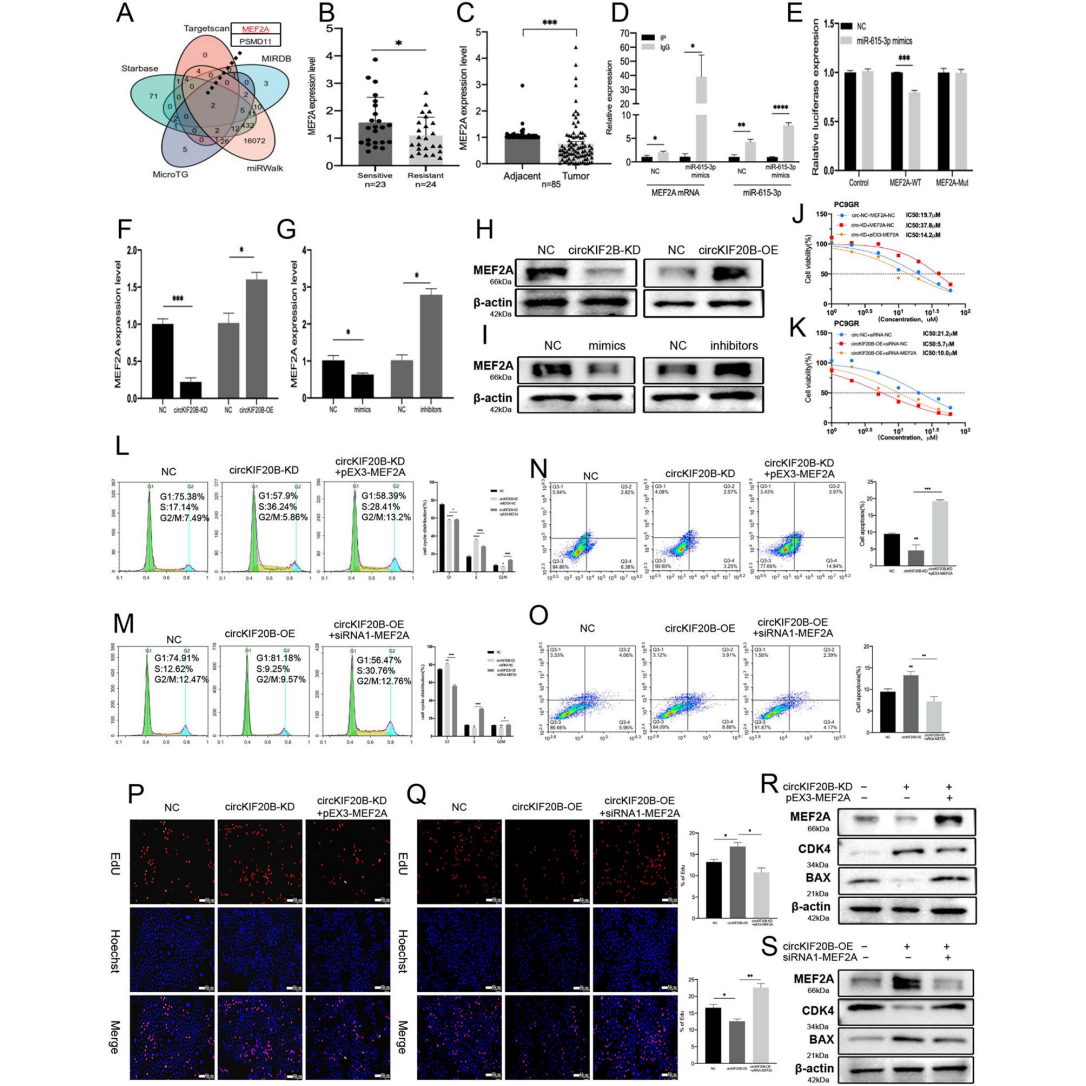
CircKIF20B regulated gefitinib resistance and cell proliferation by MEF2A. **A** The PSMD11 and MEF2A were predicted as the downstream target of miR-615-3p from 5 databases. **B** The expression of MEF2A in serum exosomes of gefitinib-resistant and sensitive patients (n = 24/23) as measured by qRT-PCR, with β-actin serving as an internal reference. **C** The expression of MEF2A in the NSCLC tissues and matched normal tissues (n = 85). **D** MEF2A and miR-615-3p expression of the anti-Ago2 group was detected by qRT-PCR in the complex from PC9 lysates co-immunoprecipitation, with IgG group serving as the control. **E** The luciferase activity of HEK293T cell co-transfected with WT or MUT vectors and the mimics of miR-615-3p. **F** and **H** The RNA and protein expressions of MEF2A using qRT-PCR and Western blotting in the circKIF20B-KD and circKIF20B-OE groups, with the cells transfected with LV3-NC and GV689-NC serving as control. **G** and **I** The RNA and protein expressions of MEF2A in PC9GR transfected with miR-615-3p mimics or inhibitors were detected using qRT-PCR and Western blotting, the cells transfected with mimics-NC or inhibitor-NC serving as control. **J** The gefitinib IC50 values of PC9GR co-transfected LV3-NC and pEX3-NC, PC9GR circKIF20B-KD transfected pEX3-NC, PC9GR circKIF20B-KD transfected pEX3-MEF2A were detected by CCK-8 assay. **K** The IC50 values of gefitinib for PC9GR co-transfected GV689-NC and siRNA-NC, PC9GR circKIF20B-OE transfected siRNA-NC, circKIF20B-OE groups transfected siRNA1-MEF2A were detected using CCK-8 assay, respectively. **L** and **N** The cell cycle and apoptosis assays of PC9GR co-transfected with LV3-NC and pEX3-NC, the circKIF20B-KD groups transfected with pEX3-NC, and the circKIF20B-KD groups transfected with pEX3-MEF2A. **M** and **O** The cell cycle and apoptosis assays of PC9GR co-transfected with GV689-NC and siRNA-NC, the circKIF20B-OE groups transfected with siRNA-NC, and the circKIF20B-OE groups transfected with siRNA1-MEF2A. **P** and **Q** EdU analyzed the cell proliferation of the abovementioned groups. **R** and **S** The MEF2A, CDK4, and BAX protein expression of the abovementioned groups were analyzed by Western blotting

The target genes of miR-615-3p were predicted using 5 public databases (i.e., Targetscan, MIRDB, Starbase, MicroT, and miRWalk). Two potential genes (*MEF2A and PSMD11*) were screened after taking the intersection (Fig. 5A). The expression of MEF2A and PSMD11 in NSCLC was detected with reference to the TCGA database. As shown in Figures S2H and S2I, the PSMD11 expression was significantly increased, whereas the MEF2A expression was significantly decreased. Correlation analysis indicated that MEF2A was significantly negatively correlated with miR-615-3p, unlike PSMD11 (Fig. S2L and S2M). Furthermore, ROC curves illustrated that MEF2A and PSMD11 possessed significant diagnostic efficacy for NSCLC (Fig. S2J and S2K). In the lung cancer group from TCGA, high expression of MEF2A was significantly associated with increased DSS and PFI of patients (Fig. S2O and S2P). The qRT-PCR results for the clinical tissues were consistent with the database results (n = 85) (Fig. 5C), and MEF2A was significantly downregulated in the serum exosomes of patients with gefitinib resistance (n = 23/24) (Fig. 5B). These results suggest that MEF2A may be a tumor suppressor gene. Therefore, MEF2A is more likely to be the target gene for miR-615-3p. RIP assay showed that MEF2A mRNA was significantly upregulated in the Ago2 group than in the control group (Fig. 5D), which proved the interaction between miR-615-3p and MEF2A. The binding site of miR-615-3p with MEF2A was verified by a dual-luciferase reporter assay (Fig. 5E and Fig. S2N). The results of both PCR and Western blotting indicated that circKIF20B regulated the expression of MEF2A and that there was a positive correlation (Fig. 5F H). Moreover, miR-615-3p directly regulated the expression of MEF2A, which was negatively correlated (Fig. 5G and I). After determining the interaction between miR-615-3p and MEF2A, the role of MEF2A in regulating gefitinib resistance was explored. Three siRNAs against different targets were employed to interfere with MEF2A. The results from PCR and Western blotting showed that the knockdown effect of siRNA-1 was highest (Fig. S1G and S1I). Similarly, the overexpression plasmid effectively increased the expression of MEF2A (Fig. S1H and S1J). Subsequently, a CCK-8 assay was performed, which revealed that interference with the MEF2A expression increased the IC50 value of PC9GR for gefitinib, whereas its overexpression significantly decreased the IC50 value (Fig. S1L). Thus, the interaction between miR-615-3p and MEF2A was clarified, and MEF2A was established to inhibit the progression of gefitinib resistance.

### CircKIF20B regulates gefitinib resistance and cell proliferation through the miR-615-3p/MEF2A axis

Four experimental rescue groups were designed to investigate whether circKIF20B regulates gefitinib resistance and cell proliferation via the miR-615-3p/MEF2A axis. The circKIF20B-KD group was transfected with the inhibitors of miR-615-3p or the overexpression vector of MEF2A, and the control group LV3-NC was transfected with the inhibitors-NC or pEX3-NC. The circKIF20B-OE group was transfected with the mimics of miR-615-3p or the siRNA-1 of MEF2A, and the control group GV689-NC was transfected with mimics-NC or siRNA-NC. EdU assay showed that the hyperproliferative effects of circKIF20B-KD were reversed by the inhibitors of miR-615-3p or by the overexpression of MEF2A (Figs. 4O and 5P). Conversely, the poor proliferation caused by the overexpression of circKIF20B was rescued by the mimics of miR-615-3p or by the siRNA-1 of MEF2A (Figs. 4P and 5Q). Rescue experiments also affected the resistance of the PC9GR cell line to gefitinib. The increase in the IC50 value caused by the knockdown of circKIF20B was reversed by the inhibitors of miR-615-3p or by the overexpression of MEF2A (Figs. 4H and 5 J), and the decrease in IC50 value caused by the overexpression of circKIF20B was reversed by the mimics of miR-615-3p or by the siRNA-1 of MEF2A (Figs. 4I and 5 K). The occurrence and development of cell proliferation and drug resistance were closely related to the effects on the cell cycle and apoptosis, and circKIF20B was demonstrated to regulate CDK4 and BAX. Therefore, cell cycle analysis and apoptosis assay were added to the experimental rescue groups. As shown in Fig. 4J M, 5 L, and 5 N, the cell cycle progression was accelerated and apoptosis was inhibited in the circKIF20B-KD group, as determined by flow cytometry, which was reversed by downregulating miR-615-3p or upregulating MEF2A. Meanwhile, the protein level of CDK4, BAX, and MEF2A was detected by Western blotting. As shown in Figs. 4Q and 5R, the expression of MEF2A and BAX were downregulated while that of CDK4 was upregulated in the circKIF20B-KD group. However, downregulating miR-615-3p or upregulating MEF2A reversed the expression level of these proteins when compared with those in the circKIF20B-KD group. Moreover, the cell cycle arrest and the high apoptosis rate in the circKIF20B-OE group were reversed by upregulating miR-615-3p or decreasing the expression of MEF2A (Fig. 4K N, 5 M, and 5O). In addition, Western blotting showed consistent trends in terms of protein levels, supporting the above results (Figs. 4R and 5 S). Cumulative results confirm that circKIF20B acts as a ceRNA to regulate the resistance to gefitinib and cell proliferation via the miR-615-3p/MEF2A axis in NSCLC.

### CircKIF20B regulates cell oxidative phosphorylation via MEF2A

**Fig. 6:**
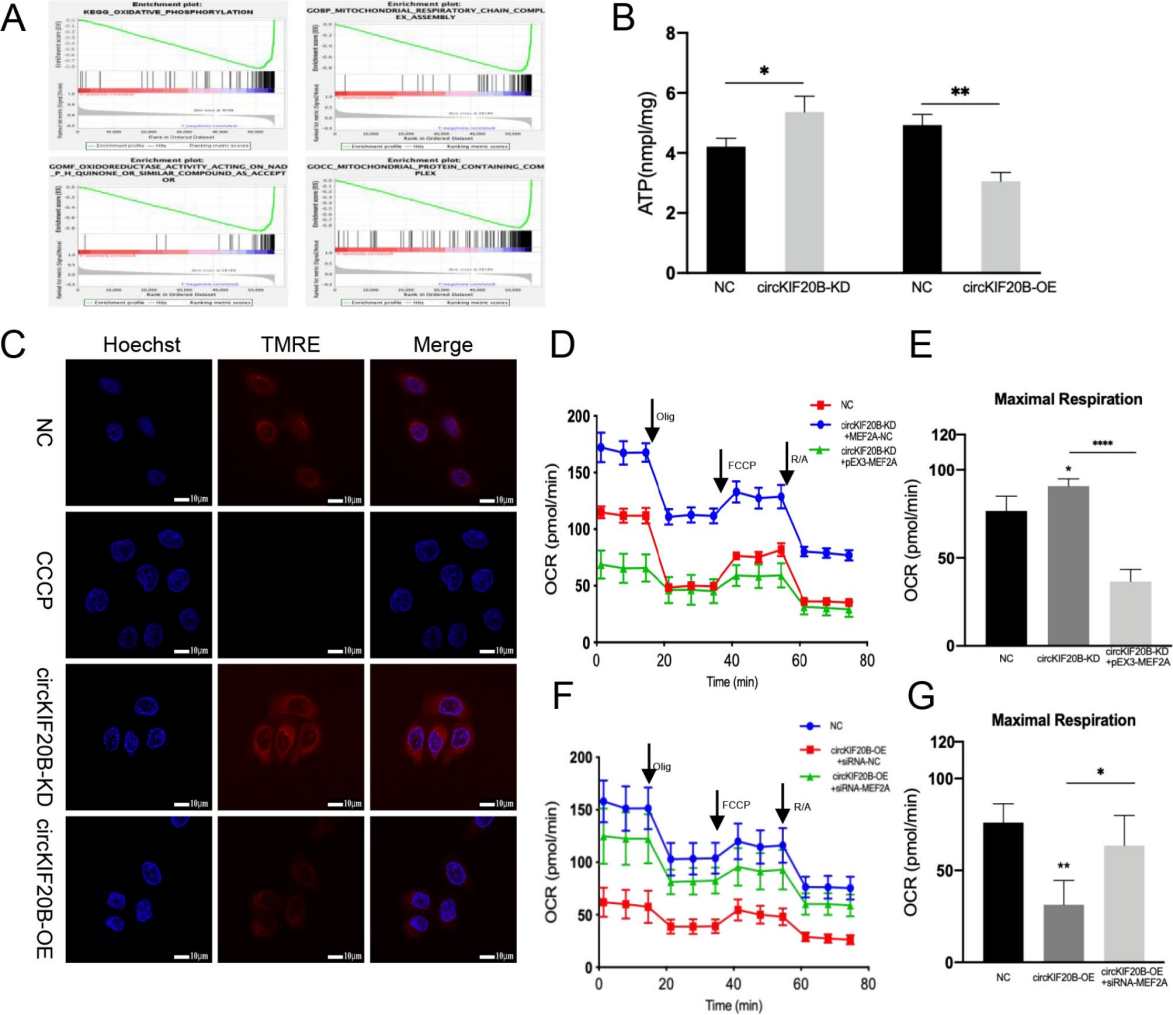
CircKIF20B regulated mitochondrial function and OXPHOS by MEF2A in NSCLC. **A** GSEA analysis found that the expression of MEF2A was significantly negatively correlated with mitochondrial function and OXPHOS. **B** ATP assay of circKIF20B-KD cells and circKIF20B-OE cells. **C** Mitochondrial membrane potential assay of circKIF20B-KD cells and circKIF20B-OE cells by TMRE. CCCP is a positive control. **D** and **E** The OCR analysis of PC9GR cells co-transfected with LV3-NC and pEX3-NC, circKIF20B-KD cells transfected with pEX3-NC, and circKIF20B-KD cells transfected with pEX3-MEF2A via Seahorse. **F** and **G** The OCR analysis of PC9GR cells co-transfected with GV689-NC and siRNA-NC, circKIF20B-OE cells transfected with siRNA-NC, and circKIF20B-OE cells transfected with siRNA1-MEF2A via Seahorse

Gene set enrichment analysis (GSEA) analysis showed that MEF2A was significantly negatively correlated with mitochondrial respiratory chain and OXPHOS (Fig. 6A). Therefore, we further investigated whether circKIF20B could regulate the OXPHOS function of NSCLC via MEF2A. The analysis of the ATP concentration revealed that the circKIF20B-KD group had a significantly higher ATP content than the control group, whereas the ATP content of the circKIF20B-OE group was remarkably lower than that of the control group (Fig. 6B). Moreover, TMRE analysis showed that the knockdown of circKIF20B enhanced the mitochondrial membrane potential of the cells, whereas the overexpression of circKIF20B reduced this potential. The CCCP group acted as the positive control (Fig. 6C). The results of Seahorse XF extracellular metabolism analysis showed that the OCR of the circKIF20B-KD group was significantly increased when compared with that of the control group, and this effect was reversed by overexpressing MEF2A (Fig. 6D and E). Meanwhile, the decrease in OCR caused by circKIF20B overexpression was rescued by siRNA of MEF2A (Fig. 6F and G). Collectively, these results reveal that circKIF20B regulates the mitochondrial energy metabolism by MEF2A in NSCLC.

### Upregulated exosomal circKIF20B restores the gefitinib sensitivity of recipient cells

**Fig. 7:**
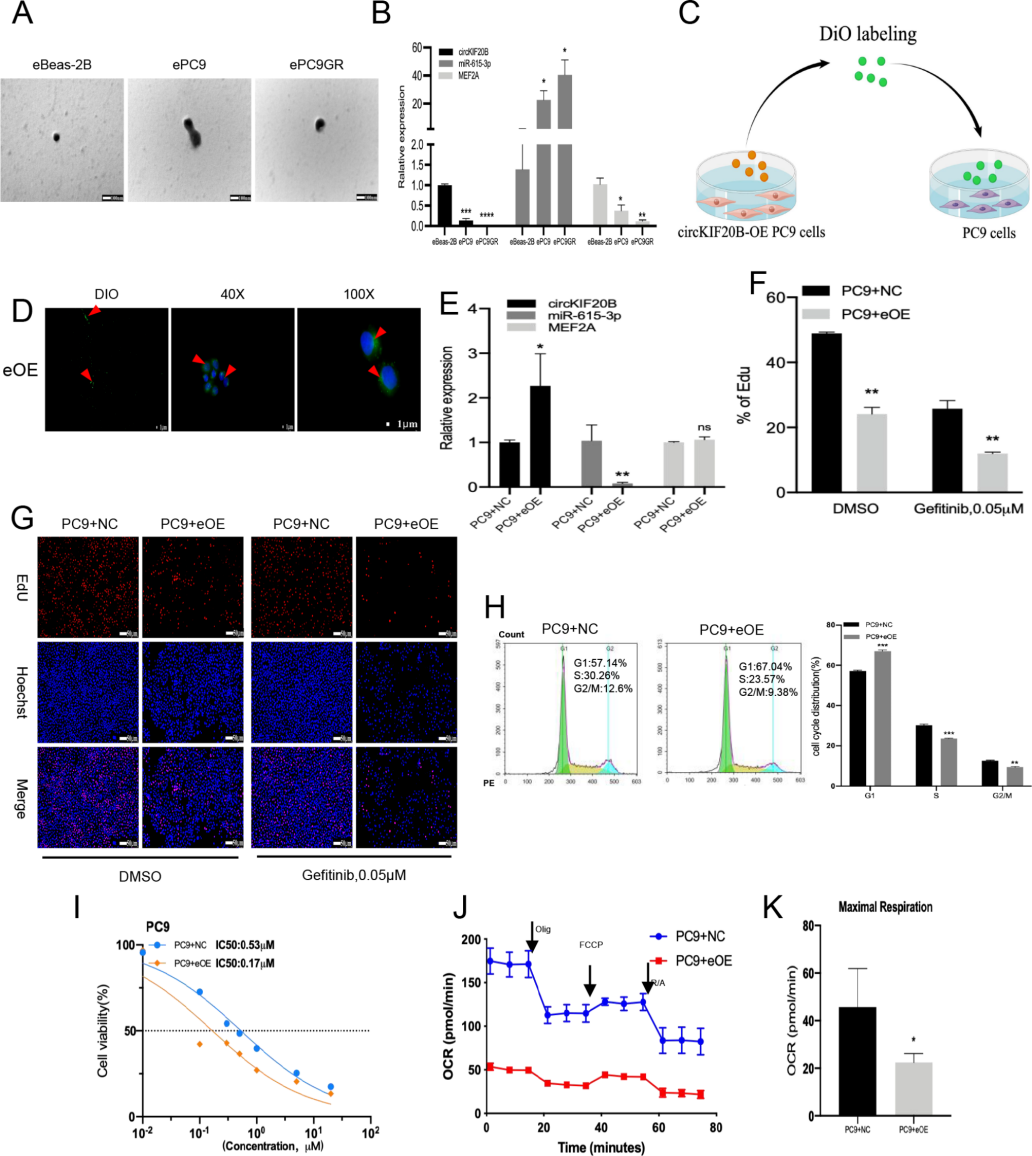
Exosome-derived circKIF20B transfers the malignant state and gefitinib resistance of NSCLC in vitro. **A** The identification of exosomes extracted from Beas-2B, PC9, and PC9GR by electron microscopy. **B** The expression of circKIF20B, miR-615-3p, and MEF2A in exosomes of Beas-2B, PC9, and PC9GR, as detected by qRT-PCR. **C** The DiO-labeled exosomes extracted from the circKIF20B-OE PC9 cells were co-cultured with PC9 for 24 h. **D** Dio-labeled overexpressed circKIF20B PC9 cell-derived exosomes were endocytosed by the recipient PC9 cells. **E** The expression of circKIF20B, miR-615-3p, and MEF2A in the PC9 cells co-cultured with exosomes from the circKIF20B-OE cells, as measured by qRT-PCR. **F** and **G** EdU assay of PC9 cells co-cultured with the exosomes (without gefitinib or with 0.05µM gefitinib). **H** Cell cycle assay of PC9 cells co-cultured with exosomes from circKIF20B-OE PC9 cells. **I** Gefitinib IC50 values of PC9 cells co-cultured with exosomes from circKIF20B-OE PC9 cells. **J** and **K** The OCR analysis of PC9 cells co-cultured with the exosomes from circKIF20B-OE PC9 cells

Extracellular vesicles have been regarded as essential mediators of intercellular communication. In this study, the exosomes were extracted from the supernatants of Beas-2B, PC9, and PC9GR and named eBeas-2B, ePC9, and ePC9GR (Fig. 7A). As shown in Fig. 7B, the expression of circKIF20B in ePC9 and ePC9GR was significantly lower than that of eBeas-2B, the expression of circKIF20B in the ePC9GR was lower than that of ePC9, the expression of miR-615-3p was negatively correlated with circKIF20B, and the expression of MEF2A was positively correlated. This correlation perfectly agrees with our previous qRT-PCR results (Fig. 1I J). The exosomes were then extracted from the cell supernatants of the circKIF20B-OE cells and eOE. As shown in Fig. 7C, the eOE were labeled with DiO and added to the PC9 for 24 h co-culturing. The exosomes were observed to enter the cytoplasm of the target cells (Fig. 7D). In the PC9 co-cultured with eOE, qRT-PCR showed that the expression of circKIF20B was dramatically increased, while that of miR-615-3p was decreased (Fig. 7E). CCK-8 analysis indicated that the PC9 co-culture with eOE significantly decreased the IC50 value of gefitinib (Fig. 7I). The EdU assay showed that the proliferative ability was significantly decreased when the PC9 was co-cultured with eOE with or without gefitinib treatment (Fig. 7F and G). Notably, the same phenomenon was observed in the cell cycle assay. In the eOE group, there was an increase in the G1 phase and a decrease in the S phase. The cell cycle was significantly arrested (Fig. 7H). Interestingly, as seen in Fig. 7J K, exosome-derived circKIF20B significantly reduced the OXPHOS in the target cells. These findings suggest that overexpressed circKIF20B not only changes the cell cycle, apoptotic properties, and OXPHOS of parental cells but also affects the proliferative properties and drug resistance of recipient cells by loading the upregulated circKIF20B exosomes.

### Dysregulated circKIF20B affects the process of NSCLC in vivo

**Fig. 8:**
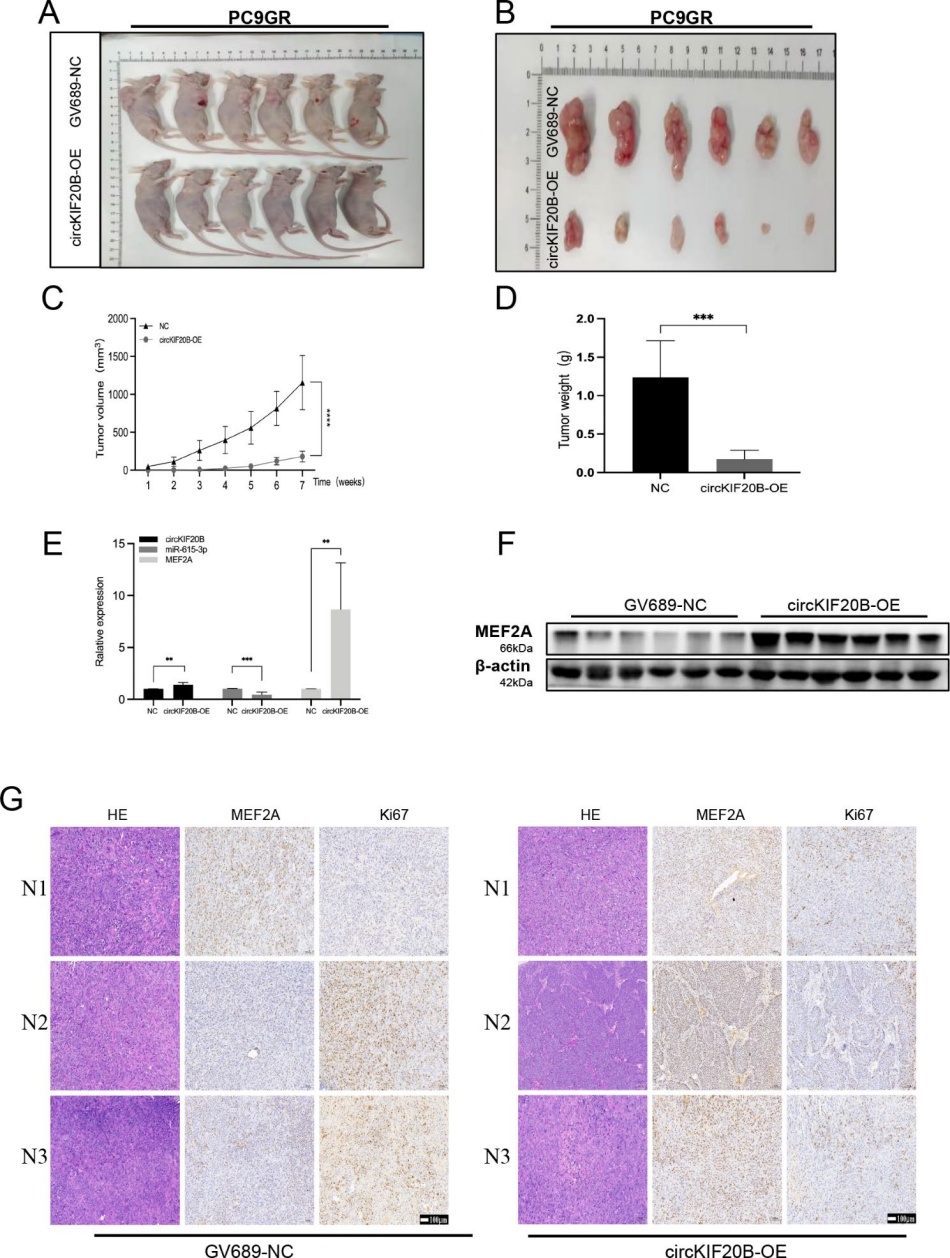
CircKIF20B acts as a tumor suppressor in NSCLC in vivo. **A** and **B** Images of the xenograft tumors from the circKIF20B-OE group, the GV689-NC, served as the control. **C** and **D** Growth curves and weight analysis of tumors from the circKIF20B-OE and GV689-NC groups. **E** The expression of circKIF20B, miR-615-3p, and MEF2A in xenograft tumors from the circKIF20B-OE and GV689-NC groups. **F** The protein expression of MEF2A in the circKIF20B-OE groups in vivo. **G** IHC and HE is staining of the tumor sections in the circKIF20B-OE group with MEF2A and Ki67 antibodies

To evaluate the role of dysregulated circKIF20B in the development of NSCLC, the circKIF20B-OE PC9GR were subcutaneously injected into Balb/c nude mice (Fig. 8A and B). The tumor volume and weight were significantly smaller and lighter in the circKIF20B-OE group than in the control group (Fig. 8C and D). qRT-PCR revealed that the RNA expressions of circKIF20B and MEF2A were upregulated and that miR-615-3p was downregulated in the circKIF20B-OE group (Fig. 8E). Western blotting indicated that the protein expression of MEF2A was upregulated (Fig. 8F), which is in line with the previous findings. IHC showed that the expression of MEF2A was upregulated, while that of Ki67 was downregulated in the circKIF20B-OE group (Fig. 8G). Together, these results demonstrate that circKIF20B is a meaningful tumor suppressor in NSCLC *in vivo.*

## Discussion

Gefitinib, as the first-line treatment for EGFR-mutated NSCLC significantly improves patients’ survival [49], albeit acquired resistance strongly limits the long-term clinical efficacy of gefitinib. In the past few years, some scientists have studied the resistance mechanism of gefitinib and found that gene mutation is the leading cause of gefitinib resistance [50]. Although some targeting genetic mutations EGFR-TKIs, such as osimertinib and afatinib, have demonstrated reliable results in second-line therapy [51, 52], their vast cost and secondary resistance have caused severe harm to the patients. Therefore, exploring new gefitinib resistance mechanisms is urgent. This study revealed a novel circRNA, circKIF20B, that was significantly downregulated in the gefitinib-resistant cellular exosomes. The expression of circKIF20B was decreased in both the serum exosomes of patients with gefitinib resistance and the tumor tissues of patients with NSCLC. This study sheds light on how exosome-derived circKIF20B regulates the cell cycle, proliferation, apoptosis, and OXPHOS to affect gefitinib resistance. This discovery is novel and differs from conventional resistance mechanisms.

This study demonstrated the advantages of exosomal circKIF20B as an alternative liquid biopsy candidate for diagnosing and treating gefitinib resistance in NSCLC in terms of the following points: first, characterization experiments signified that the circular structure made circKIF20B more resistant to ACTD and RNase R. The lipid membrane encapsulation of the exosomes reduced the risk for degradation [53]. Moreover, the stable expression of circKIF20B in the human tissues and serum exosomes was verified in this study, and the expression in the tissues was observed to be consistent with that in the serum exosomes. This finding suggests that exosomal circKIF20B is highly specific and stable as a noninvasive biopsy biomarker and a candidate for gene therapy. In addition, the results from gain and loss experiments implied that the overexpression of circKIF20B significantly decreased the IC50 value of gefitinib and the proliferation of the NSCLC cells. Contrarily, the downregulation of circKIF20B enhanced the gefitinib resistance and cell proliferation. This finding provides strong scientific evidence for diagnosing, monitoring, and treating acquired drug resistance. Finally, the recipient cells were intervened with exosomes extracted from NSCLC cell lines with artificially upregulated circKIF20B expression to mimic biological communication between the cancer cells. After exosomes loaded with upregulated circKIF20B entered the target cells, exosomal circKIF20B, as a unique gene therapy modality, suppressed target cells’ gefitinib resistance and proliferation properties. Exosome-derived circKIF20B appears to hold considerable potential in engineered therapy for reversing gefitinib resistance, which agrees with the findings of Huang’s research [54].

Mechanistically, this study found that circKIF20B was significantly enriched in the cytoplasm, suggesting the potential of circKIF20B as a miRNA sponge to function as a ceRNA [55]. MiR-615-3p was screened out by bioinformatics analysis, luciferase reporter gene, and RNA pulldown. CircKIF20B has a natural binding ability to the miR-615-3p-response element, which can downregulate the inhibitory effect of miR-615-3p on target mRNA. Notably, miR-615-3p appeared to be a bidirectionally regulated tumor-associated molecule. Liu found that circITCH increases bortezomib resistance in multiple myeloma by upregulating miR-615-3p [56]. Lei found that miR-615-3p acts as an oncogenic factor that promotes EMT and metastasis in breast cancer by regulating the PICK1/TGFBRI axis [57]. However, Professor Pan’s research reported that miR-615-3p is one of the critical factors in the fight against erlotinib resistance [58]. In our study, the expression of miR-615-3p was significantly upregulated in both the serum exosomes of 23 patients with gefitinib resistance and 85 NSCLC tissues, which is consistent with the miRNA-seq data of the TCGA; moreover, its expression was negatively correlated with the expression of circKIF20B. The IC50 value of the gefitinib assay indicated that miR-615-3p could significantly promote the level of gefitinib resistance. In corroboration with previous reports, we found that circKIF20B acted as a tumor suppressor gene in the progression of gefitinib resistance via binding the oncogene miR-615-3p.

Mature miRNAs bind to the 3’ untranslated region (UTR) of target mRNAs and trigger mRNA degradation or translational repression. We found that the 3’UTR of MEF2A can directly bind to the MRE of miR-615-3p through bioinformatics analysis, dual-luciferase reporter gene assay, and RIP. To date, this study is the first to report the endogenous existence of the circKIF20B/miR-615-3p/MEF2A signaling axis in NSCLC cells. MEF2A has been reported to be related to the cell cycle and apoptosis [59], as also validated by Western blotting in our study. The interaction of the MEF2 family with EGFR inhibits cell proliferation in drug-resistant metastatic colorectal cancer [60]. In our study, the expression of MEF2A was significantly reduced in the NSCLC tissues and the serum exosomes of patients with gefitinib resistance, which is consistent with the RNA-seq data in TCGA. The qRT-PCR and Western blotting indicated that circKIF20B and miR-615-3p could independently regulate the transcriptional and translational expression of MEF2A. Moreover, we found that circKIF20B regulates the cell cycle, proliferation, and apoptosis in gefitinib resistance via the miR-615-3p/MEF2A axis.

GSEA showed that the expression of MEF2A was significantly negatively correlated with mitochondrial metabolism and OXPHOS. A past study showed that miRNA could enhance the mitochondrial function and activate OXPHOS by downregulating MEF2A [61], while tumor-related genes enhance OXPHOS through metabolic reprogramming to inhibit sensitivity to TKI drugs [62]. Moreover, it is interesting that Gong’s research showed that circPUM1 accelerated the development of cancer by increasing the mitochondrial membrane potential of the esophageal squamous cell carcinoma cells and enhancing the function of OXPHOS [63]. This finding prompted an investigation of whether circKIF20B could regulate the mitochondrial function and OXPHOS in the NSCLC cells via MEF2A. As we all know that cancer cells have specific energy metabolism, aerobic glycolysis. It can support cancer cells surviving the same as OXPHOS. It has been reported that tumor tissues have an increased glucose metabolism and lactic acid production. However, the availability of glutamine is low in both normal and tumor tissues. Meanwhile, tumor tissues need more glucose as the main carbon source in the TCA cycle compared to healthy tissues. All the evidence demonstrated that glucose carbon is essential for tumor formation [64]. Both aerobic glycolysis and oxidative phosphorylation are involved in cancer progression. It has been reported that upregulating MEF2A inhibited the function of mitochondria [65]. And MEF2A played roles in muscle cells and drove DIK1-Dio3 cluster to inhibit mitochondrial activity. Meanwhile, MEF2A can regulate glycolytic versus oxidative myofiber development [66]. In our study, we detected that the level of ATP was increased in circKIF20B-KD group and decreased in circKIF20B-OE group compared with the NC group. It suggested that circKIF20B may perform a critical role in cancer cells’ energy metabolism. To further confirm the role of circKIF20B and investigate the potential mechanism, we regulated the expression of MEF2A and measured the OCR and Mitochondrial membrane potential. Our result showed that circKIF20B significantly inhibited cellular ATP synthesis, mitochondrial membrane potential, and cellular OXPHOS. However, MEF2A could reverse these effects. Furthermore, exosomes loaded with dysregulated circKIF20B could significantly alter the OXPHOS in the target cells.

This result implied that circKIF20B/miR-615-3p/MEF2A signal axis reduced the gefitinib resistance by arresting the cell cycle, promoting cell apoptosis, and altering the cellular energy metabolism. This axis is a novel mechanism that differs from other conventional drug resistance models. The overexpression of circKIF20B could not only alter the energy metabolism and growth ability of parent cells but also be endogenously loaded by exosomes to inhibit the proliferation and gefitinib resistance of the recipient cells.

## Conclusions

In summary, a novel gefitinib-resistance mechanism was proposed for the first time in this study. Exosomal circKIF20B regulated the miR-615-3p/MEF2A axis to inhibit gefitinib resistance and NSCLC proliferation by arresting the cell cycle, promoting apoptosis, and reducing mitochondrial OXPHOS. The unreported exosome-derived circRNA, circKIF20B, holds the potential to serve as a noninvasive and stable candidate for detecting and treating gefitinib resistance. This study thus explored a novel mechanism of acquired resistance to gefitinib, likely to provide new targets and research directions for detecting and treating NSCLC.

## Electronic supplementary material

Below is the link to the electronic supplementary material.

Additional file 1 (Table S1-S4).

Additional file 2 (Figure S1-S2).

## Data Availability

All data generated or analyzed during this study are included in this published article and its Additional Files.

## List of Abbreviations

ACTD: Actinomycin-D
CCCP: Carbonyl cyanide m-chlorophenyl hydrazone
DiO: 3,3-Dioctadecyloxacarbocyanine perchlorate
EdU: 5-Ethynyl-20-deoxyuridine
FCCP: Carbonyl cyanide-4-(trifluoromethoxy) phenylhydrazone
GSEA: Gene set enrichment analysis
MEF2A: Myocyte-specific enhancer factor 2 A
PSMD11: Proteasome 26 S Subunit, Non-ATPase 11
RNase R: Ribonuclease R
ROC: Receiver operating characteristic curve
TCGA: The cancer genome atlas
TMRE: Tetramethylrhodamine ethyl ester
