# Supplementary material for: Exosome-derived circKIF20B suppresses gefitinib resistance and cell proliferation in non-small cell lung cancer
Source: Cancer Cell Int. 2023 Jul 2;23:129. doi: 10.1186/s12935-023-02974-y (PMC10316567; doi:10.1186/s12935-023-02974-y)
Supplement: Supplementary file 1 — Additional file 1 (Table S1-S4). [file 12935_2023_2974_MOESM1_ESM.docx]

**Additional file 1**

**This file includes:**

**Table S1.** A part of the differential expression profile in exosome-derived circRNA-seq

**Table S2.** List of relevant database URLs

**Table S3.** List of relevant sequences

**Table S4.** List of relevant antibodies

**Table S1. A part of differential expression profile in exosome-derived circRNA-seq**

| **Circbase ID** | **PC9-1** | **PC9-2** | **PC9-3** | **PC9GR-1** | **PC9GR-2** | **PC9GR-3** |
| --- | --- | --- | --- | --- | --- | --- |
| hsa_circ_0129482 | 0.0000 | 0.0000 | 0.1989 | 0.0000 | 1.9747 | 4.9748 |
| hsa_circ_0006867 | 0.0000 | 0.0000 | 0.0000 | 0.5944 | 0.6582 | 0.7107 |
| hsa_circ_0008901 | 0.0000 | 0.0000 | 0.7956 | 3.5663 | 2.3038 | 11.0155 |
| hsa_circ_0035296 | 0.2593 | 0.0000 | 0.0000 | 1.1888 | 0.6582 | 3.5534 |
| hsa_circ_0073172 | 0.0000 | 0.2629 | 0.0000 | 0.0000 | 1.6456 | 2.4874 |
| hsa_circ_0005060 | 0.0000 | 0.2629 | 0.0000 | 0.0000 | 3.2911 | 0.7107 |
| hsa_circ_0059482 | 0.0000 | 0.0000 | 0.1989 | 0.0000 | 1.9747 | 1.7767 |
| hsa_circ_0008482 | 0.0000 | 0.0000 | 0.1989 | 1.7832 | 0.6582 | 1.4214 |
| hsa_circ_0004503 | 0.0000 | 0.0000 | 0.3978 | 2.9719 | 3.2911 | 0.0000 |
| hsa_circ_0130074 | 0.0000 | 0.0000 | 0.1989 | 0.0000 | 2.3038 | 1.0660 |
| chr17_15508536_15519123_- | 0.0000 | 0.0000 | 0.1989 | 0.5944 | 0.9873 | 1.7767 |
| hsa_circ_0102765 | 0.0000 | 0.0000 | 0.1989 | 0.0000 | 1.9747 | 1.0660 |
| hsa_circ_0117271 | 0.0000 | 0.2629 | 0.0000 | 1.7832 | 1.6456 | 0.0000 |
| hsa_circ_0125415 | 0.5186 | 0.0000 | 0.0000 | 0.5944 | 3.2911 | 1.0660 |
| hsa_circ_0066888 | 0.2593 | 0.2629 | 0.0000 | 0.5944 | 3.2911 | 1.0660 |
| chr2_236817381_236877267_+ | 0.5186 | 0.0000 | 0.0000 | 0.5944 | 0.9873 | 3.1981 |
| hsa_circ_0107900 | 0.2593 | 0.0000 | 0.1989 | 0.0000 | 3.6202 | 0.7107 |
| hsa_circ_0007772 | 0.5186 | 0.0000 | 0.1989 | 1.1888 | 1.9747 | 2.8427 |
| hsa_circ_0006289 | 0.5186 | 0.0000 | 0.0000 | 2.3776 | 0.0000 | 2.1320 |
| hsa_circ_0000958 | 0.5186 | 0.5257 | 0.0000 | 3.5663 | 2.6329 | 1.7767 |
| hsa_circ_0001565 | 0.7778 | 0.0000 | 0.1989 | 4.7551 | 0.0000 | 2.1320 |
| hsa_circ_0006874 | 0.5186 | 0.0000 | 1.1933 | 1.1888 | 3.6202 | 3.9087 |
| hsa_circ_0073435 | 0.2593 | 1.5772 | 0.5967 | 0.5944 | 6.9114 | 2.4874 |
| hsa_circ_0004365 | 1.2964 | 0.2629 | 3.3811 | 0.0000 | 0.3291 | 0.0000 |
| hsa_circ_0069982 | 0.2593 | 3.4172 | 1.9889 | 0.5944 | 0.0000 | 0.0000 |
| hsa_circ_0001485 | 3.1113 | 4.9943 | 1.1933 | 0.0000 | 0.0000 | 0.7107 |
| hsa_circ_0001564 | 1.2964 | 1.0514 | 3.3811 | 0.5944 | 0.0000 | 0.0000 |
| hsa_circ_0003592 | 0.0000 | 2.3657 | 3.3811 | 0.5944 | 0.0000 | 0.0000 |
| hsa_circ_0007142 | 2.8521 | 1.3143 | 1.7900 | 0.5944 | 0.0000 | 0.0000 |
| hsa_circ_0058040 | 4.6670 | 1.3143 | 7.9556 | 0.0000 | 0.0000 | 1.0660 |
| hsa_circ_0000347 | 1.5557 | 2.8914 | 0.9944 | 0.0000 | 0.3291 | 0.0000 |
| hsa_circ_0114896 | 1.0371 | 1.8400 | 2.7844 | 0.0000 | 0.3291 | 0.0000 |
| hsa_circ_0001750 | 2.0742 | 1.5772 | 2.1878 | 0.0000 | 0.3291 | 0.0000 |
| hsa_circ_0101710 | 2.3335 | 3.4172 | 0.1989 | 0.0000 | 0.3291 | 0.0000 |
| hsa_circ_0023942 | 4.9263 | 0.0000 | 0.9944 | 0.0000 | 0.3291 | 0.0000 |
| hsa_circ_0003787 | 0.7778 | 3.1543 | 2.7844 | 0.5944 | 0.0000 | 0.0000 |
| hsa_circ_0003511 | 2.3335 | 2.1029 | 2.3867 | 0.5944 | 0.0000 | 0.0000 |
| hsa_circ_0001746 | 3.1113 | 4.2057 | 3.7789 | 0.0000 | 0.6582 | 0.0000 |
| hsa_circ_0002058 | 1.5557 | 2.3657 | 2.9833 | 0.5944 | 0.0000 | 0.0000 |
| hsa_circ_0030067 | 0.2593 | 0.2629 | 0.7956 | 0.0000 | 0.0000 | 0.0000 |
| hsa_circ_0003310 | 4.1484 | 1.0514 | 1.3922 | 0.0000 | 0.0000 | 0.3553 |
| chr14_75245149_75249028_+ | 0.2593 | 0.5257 | 0.5967 | 0.0000 | 0.0000 | 0.0000 |
| hsa_circ_0005729 | 0.2593 | 0.5257 | 0.5967 | 0.0000 | 0.0000 | 0.0000 |
| hsa_circ_0001566 | 3.6299 | 5.2572 | 8.1545 | 0.0000 | 0.6582 | 0.3553 |
| chr2_143588766_143718339_+ | 3.1113 | 2.1029 | 2.1878 | 0.5944 | 0.0000 | 0.0000 |
| hsa_circPDS5A_013 | 3.8892 | 2.8914 | 2.1878 | 0.5944 | 0.0000 | 0.0000 |
| hsa_circ_0019079 | 3.6299 | 2.8914 | 6.5633 | 0.0000 | 0.3291 | 0.0000 |
| chr7_36445789_36456799_+ | 25.6685 | 22.0801 | 22.4745 | 0.0000 | 0.3291 | 0.0000 |

**Table S2. List of relevant database URLs**

| **Name** | **URL** |
| --- | --- |
| The cancer genome atlas (TCGA) | <https://cancergenome.nih.gov> |
| R project | https://www.r-project.org/ |
| Circbase | https://www.circrna.org |
| UCSC genome browser | https://genome.ucsc.edu |
| Circbank | https://www.circbank.cn |
| Circinteratome | https://circinteractome.irp.nia.nih.gov |
| Miranda | http://www.microrna.org/microrna/home.do |
| RNA-Hybrid | https://bibiserv.cebitec.uni-bielefeld.de/rnahybrid/ |
| Targetscan | http://www.targetscan.org |
| MIRDB | http://mirdb.org |
| Starbase | http://starbase.sysu.edu.cn |
| MicroT | www.microrna.gr/microT-v4 |
| miRWalk | http://mirwalk.umm.uni-heidelberg.de |

**Table S3. List of relevant sequences**

|  | | | |
| --- | --- | --- | --- |
| **qRT-PCR primers** | circKIF20B | Forward primer（5'-3') | GAAGAGTAATGAAATGGAGGAGATCT |
|  |  | Reverse primer（5'-3') | AAGCACTTGATCCTGTTCTACCTG |
|  | KIF20B | Forward primer（5'-3') | GCTGACTTTAAGGAGACTCTGCT |
|  |  | Reverse primer（5'-3') | GTGGCACAAATGTCTTTCGCTGC |
|  | miR-615-3p | Forward primer（5'-3') | CGTCCGAGCCTGGGTCTC |
|  |  | Reverse primer（5'-3') | AGTGCAGGGTCCGAGGTATT |
|  |  | Stem-loop primers（5'-3') | GTCGTATCCAGTGCAGGGTCCGAGGTATTCGCACTGGATACGACAAGAGG |
|  | MEF2A | Forward primer（5'-3') | AGCTGAAATAACAGAAGCTGTGTAC |
|  |  | Reverse primer（5'-3') | CCTTTCATCCATTATGCGTGTG |
|  | β-actin | Forward primer（5'-3') | CACCATTGGCAATGAGCGGTTC |
|  |  | Reverse primer（5'-3') | AGGTCTTTGCGGATGTCCACGT |
|  | U6 | Forward primer（5'-3') | GCTCGCTTCGGCAGCACATATAC |
|  |  | Reverse primer（5'-3') | AGTGCAGGGTCCGAGGTATT |
|  |  | Stem-loop primers（5'-3') | GTCGTATCCAGTGCAGGGTCCGAGGTATTCGCACTGGATACGACAAAATATGG |
|  | PCR circKIF20B  Convergent | Forward primer（5'-3') | CATCTTCAGG ATTCTGTC |
|  |  | Reverse primer（5'-3') | AGATCTGCTTTCAGATTCTG |
| **Sequences of the knockdown vectors** | circKIF20B-siRNA-1 | Forward（5'-3') | GGAGGAGAUCUAAAUGUUATT |
|  |  | Reverse（5'-3') | UAACAUUUAGAUCUCCUCCTT |
|  | circKIF20B-siRNA-2 | Forward（5'-3') | GAAAUGGAGGAGAUCUAAATT |
|  |  | Reverse（5'-3') | UUUAGAUCUCCUCCAUUUCTT |
|  | circKIF20B-LV3-NC | （5'-3') | TTCTCCGAACGTGTCACGT |
|  | miR-615-3p inhibitor | （5'-3') | AAGAGGGAGACCCAGGCUCGGA |
|  | inhibitor-NC | （5'-3') | CAGUACUUUUGUGUAGUACAA |
|  | MEF2A-siRNA-1 | Forward （5'-3') | GCUUCAACUCGCCAGGAAUTT |
|  |  | Reverse （5'-3') | AUUCCUGGCGAGUUGAAGCTT |
|  | MEF2A-siRNA-2 | Forward （5'-3') | GGACUUGUGUACUCAGCAATT |
|  |  | Reverse （5'-3') | UUGCUGAGUACACAAGUCCTT |
|  | MEF2A-siRNA-3 | Forward （5'-3') | GCAGCCAGCUCAACGUUAATT |
|  |  | Reverse （5'-3') | UUAACGUUGAGCUGGCUGCTT |
|  | MEF2A-siRNA-NC | Forward （5'-3') | UUCUCCGAACGUGUCACGUTT |
|  |  | Reverse （5'-3') | ACGUGACACGUUCGGAGAATT |
| **Sequences of the overexpression vectors** | circKIF20B-GV689 | Forward （5'-3') | CTCCCCACCATCACTTTTTAGATCTAAATGTTAAAGAGAAAATAATTG |
|  |  | Reverse （5'-3') | AACTTGGGAAATTCTTTTGTACCTCCTCCATTTCATTACTCTTCCTCTTCCG |
|  | miR-615-3p mimics | Forward （5'-3') | UCCGAGCCUGGGUCUCCCUCUU |
|  |  | Reverse （5'-3') | GAGGGAGACCCAGGCUCGGAUU |
| **Sequences of the probes** | circKIF20B FISH probe | （5'-3') | TTAGATCTCCTCCATTTCATTACTCTAACATTTAGATCTCCTCCATTTCACTCTTTAACATTTAGATCTCCTCCA |
|  | miR-615-3p FISH probe | （5'-3') | AAGAGGGAGACCCAGGCTCGGA |
|  | circKIF20B pulldown probe | （5'-3') | UUUCUCUUUAACAUUUAGAUCUCCUCCAUUUCAUUACUC |
|  | NC Biotin probe | （5'-3') | AAACAGUACUGGUGUGUAGUACGAGCUGAAGCUAC |

**Table S4. List of relevant antibodies**

| **Name** | **Brand** | **Country** | **Dilution ratio** |
| --- | --- | --- | --- |
| CD63 | Huabio, SY21-02 | China | 1˸1000 |
| TSG101 | Huabio, JJ0900 | China | 1˸1000 |
| CD9 | Huabio, SA35-08 | China | 1˸1000 |
| β-actin | Abcam, ab6276 | USA | 1˸10000 |
| MEF2A | Abcam, ab76063 | USA | WB: 1˸5000 |
|  |  | USA | IHC: 1˸250 |
| CDK4 | CST, 12790 | USA | 1˸1000 |
| BAX | CST, 5023 | USA | 1˸1000 |
| Ki67 | Proteintech, 27309-1-AP | China | IHC: 1˸2000 |
