# Supplementary material for: Exosome-derived circKIF20B suppresses gefitinib resistance and cell proliferation in non-small cell lung cancer
Source: Cancer Cell Int. 2023 Jul 2;23:129. doi: 10.1186/s12935-023-02974-y (PMC10316567; doi:10.1186/s12935-023-02974-y)
Supplement: Supplementary file 2 — Additional file 2 (Figure S1-S2). [file 12935_2023_2974_MOESM2_ESM.docx]

**Additional file 2**

**This file includes:**

**Figure S1**

**Figure S2**

**
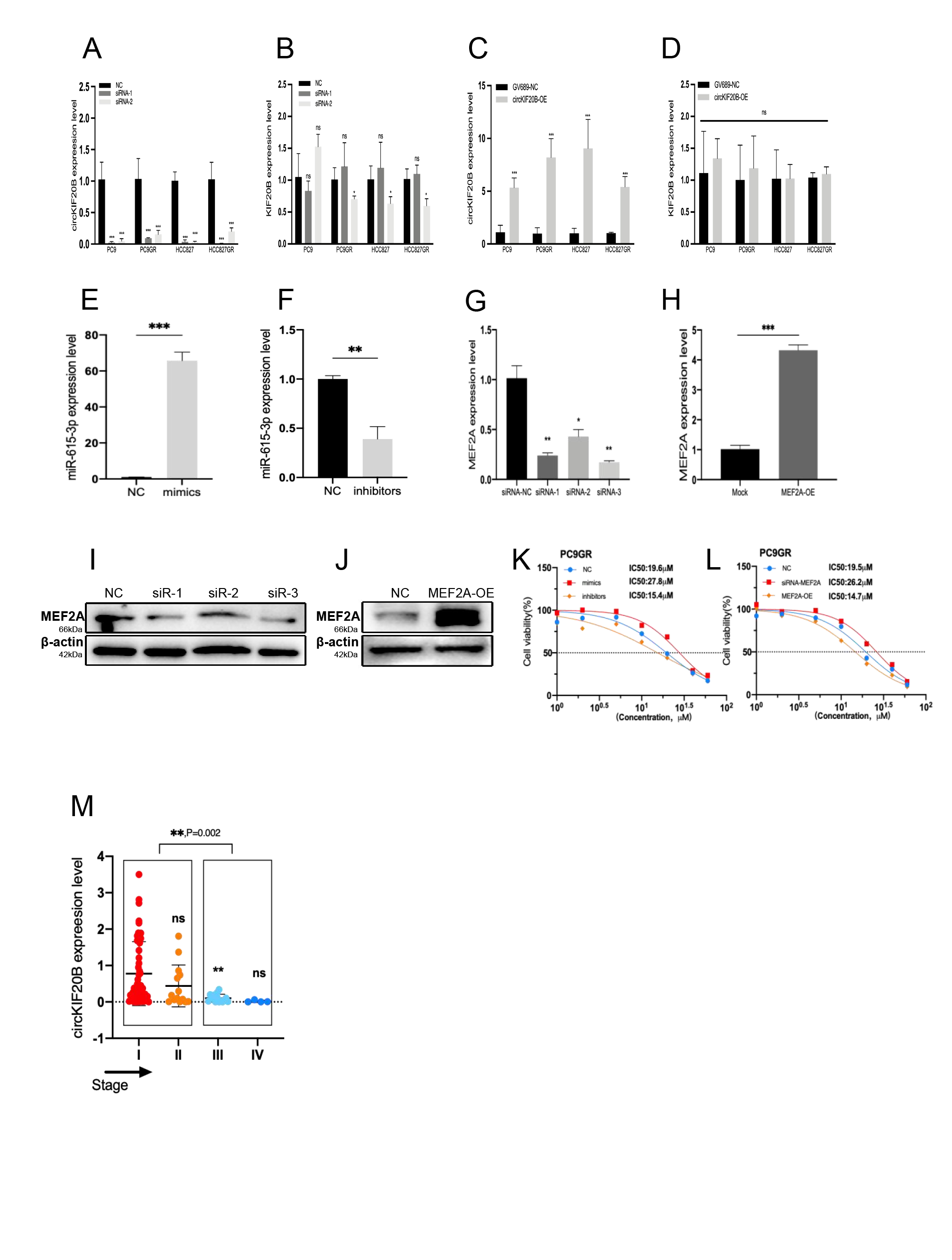
**

**Figure S1 A** and **B** Validation of siRNA knockdown efficiency were determined by qRT-PCR in four NSCLC cell lines. **C** and **D** Validation of overexpression efficiency was determined by qRT-PCR in four NSCLC cell lines. **E** and **F** Validation of miR-615-3p overexpression and knockdown efficiency was determined by qRT-PCR in PC9GR. **G**, **H**, **I**, and **J** Validation of MEF2A overexpression and knockdown efficiency was determined by qRT-PCR and western blot in PC9GR. **K** Gefitinib IC50 values of PC9GR transfected with miR-615-3p mimics or inhibitors. **L** Gefitinib IC50 values of PC9GR cells transfected with siRNA1-MEF2A or pEX3-MEF2A. **M** The relative expression of circKIF20B in a tumor stage-specific manner.

**
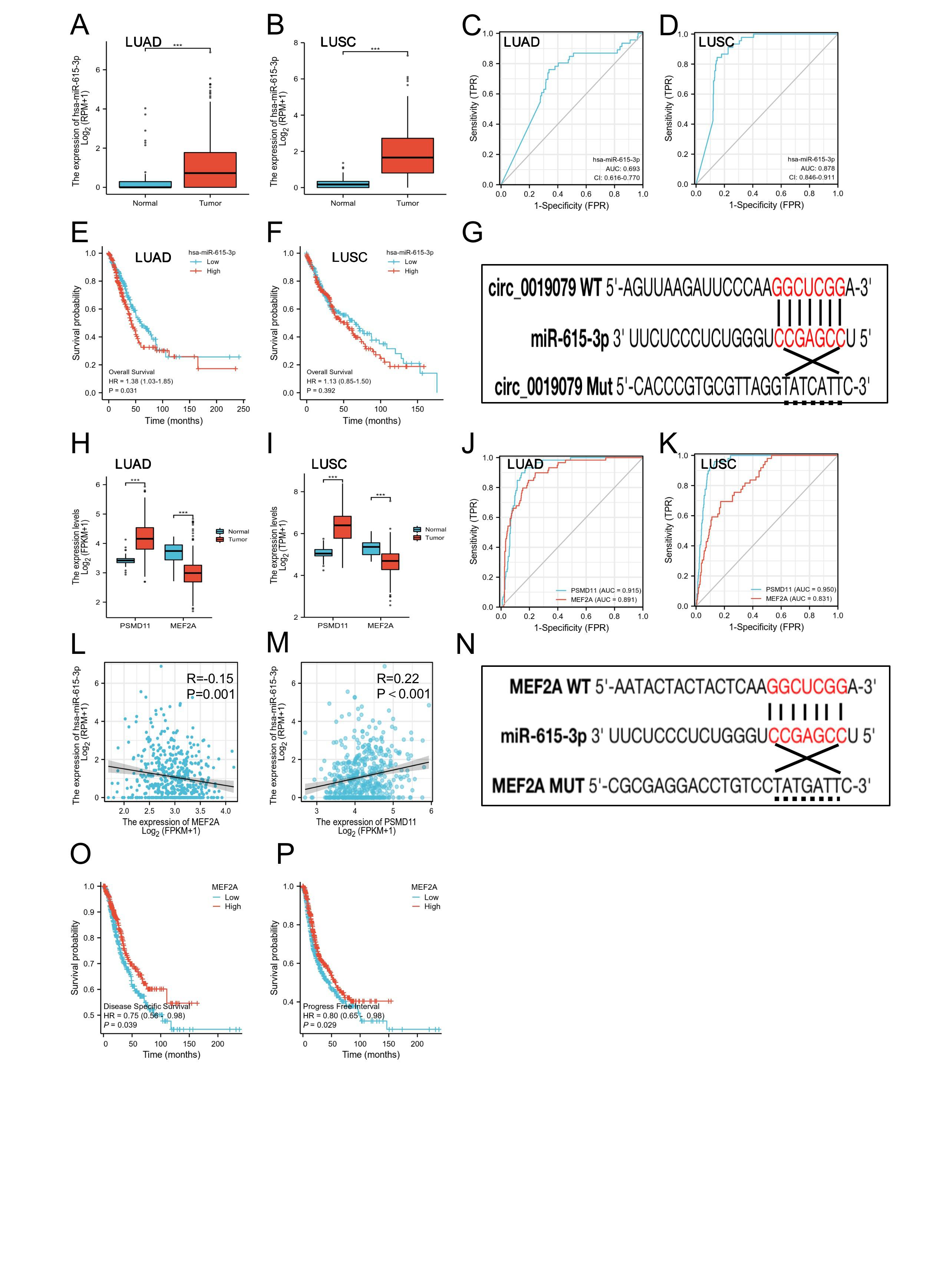
**

**Figure S2 A** and **B** The expression of miR-615-3p in LUAD and LUSC projects from TCGA. **C** and **D** The ROC curves of miR-615-3p in LUAD and LUSC projects from TCGA. **E** and **F** Survival analysis of miR-615-3p in LUAD and LUSC projects from the TCGA. **G** Binding sites of circKIF20B and miR-615-3p predicted by Circinteratome. **H** and **I** The expressions of MEF2A and PSMD11 in LUAD and LUSC projects from TCGA. **J** and **K** The ROC curves of MEF2A and PSMD11 in LUAD and LUSC projects from TCGA. **L** and **M** Correlation analysis of the expression of miR-615-3p with the expression of MEF2A, and PSMD11, respectively, from TCGA. **N** The Circinteratome predicted n Binding sites of miR-615-3p and MEF2A. **O DSS analysis of MEF2A in Lung cancer project from the TCGA. P PFI analysis of MEF2A in Lung cancer project from the TCGA.**
